# Comparative transcriptome analysis reveals key pathways and regulatory networks in early resistance of *Glycine max* to soybean mosaic virus

**DOI:** 10.3389/fmicb.2023.1241076

**Published:** 2023-10-19

**Authors:** Han Li, Jinyang Liu, Xingxing Yuan, Xin Chen, Xiaoyan Cui

**Affiliations:** ^1^College of Life Sciences, Nanjing Agricultural University, Nanjing, China; ^2^Jiangsu Key Laboratory for Horticultural Crop Genetic Improvement, Institute of Industrial Crops, Jiangsu Academy of Agricultural Sciences, Nanjing, China; ^3^College of Plant Protection, Nanjing Agricultural University, Nanjing, China

**Keywords:** soybean, soybean mosaic virus, RNA sequencing, DEGs, PPI network

## Abstract

As a high-value oilseed crop, soybean [*Glycine max* (L.) Merr.] is limited by various biotic stresses during its growth and development. *Soybean mosaic virus* (SMV) is a devastating viral infection of soybean that primarily affects young leaves and causes significant production and economic losses; however, the synergistic molecular mechanisms underlying the soybean response to SMV are largely unknown. Therefore, we performed RNA sequencing on SMV-infected resistant and susceptible soybean lines to determine the molecular mechanism of resistance to SMV. When the clean reads were aligned to the *G. max* reference genome, a total of 36,260 genes were identified as expressed genes and used for further research. Most of the differentially expressed genes (DEGs) associated with resistance were found to be enriched in plant hormone signal transduction and circadian rhythm according to Kyoto Encyclopedia of Genes and Genomes analysis. In addition to salicylic acid and jasmonic acid, which are well known in plant disease resistance, abscisic acid, indole-3-acetic acid, and cytokinin are also involved in the immune response to SMV in soybean. Most of the Ca^2+^ signaling related DEGs enriched in plant-pathogen interaction negatively influence SMV resistance. Furthermore, the MAPK cascade was involved in either resistant or susceptible responses to SMV, depending on different downstream proteins. The phytochrome interacting factor-cryptochrome-R protein module and the MEKK3/MKK9/MPK7-WRKY33-CML/CDPK module were found to play essential roles in soybean response to SMV based on protein-protein interaction prediction. Our findings provide general insights into the molecular regulatory networks associated with soybean response to SMV and have the potential to improve legume resistance to viral infection.

## 1 Introduction

*Glycine max* is a high-quality source of vegetable protein and oil, as well as a major economic crop worldwide (Liu et al., 2012). However, various pathogens and insects have a significant impact on the productivity and quality of soybean. Among the diseases, SMV (genus *Potyvirus*, family Potyviridae), is a widespread virus that causes leaf discoloration, curling, and seed mottling (Yang et al., 2014). SMV can spread from the initial site of infection to nearby cells and is transmitted by numerous aphid vectors (Irwin and Goodman, 1981), symptoms caused by some SMV strains can also be transmitted by seed (Hobbs et al., 2003). Diseases caused by SMV are widespread in major soybean production areas around the world, causing production losses of up to 10–35% each year under field conditions (Ross, 1983; Yang et al., 2013), and are the most important disease affecting global soybean production.

Similar to other plant viruses, SMV infection in field-grown soybeans has been difficult to eradicate with chemical agents (Nakano et al., 1987; Pedersen et al., 2007), and the most effective strategy for controlling this disease is the use of resistant cultivars, which is both economical and environmentally friendly. Screening and discovery of resistance genes not only provides the basis for breeding resistant germplasms, but also provides insight into the detailed mechanisms of soybean-SMV interaction. Previous studies have focused on mapping major SMV resistance QTLs (quantitative trait loci), and a number of genetic loci have been identified (Yang and Gai, 2011; Karthikeyan et al., 2018; Yuan Y. et al., 2020). *Rsv* (resistance to SMV) loci, which comprise dominant resistance (*R*) genes (Hayes et al., 2000; Gunduz et al., 2002; Liao et al., 2002; Zheng et al., 2005; Cervantes-Martinez et al., 2015), with alleles *Rsv1*, *Rsv3* and *Rsv4* have been reported to be effective against several North American SMV strains (Wen et al., 2013). Resistance to the strains from China is derived from *Rsc* loci, mapped to chromosomes 2, 13, 14, and 6 in various resistant cultivars (Ma et al., 2011; Wang D. G. et al., 2011; Zheng et al., 2014; Rui et al., 2017). Another mechanism of plant resistance to viruses is referred to as recessive resistance, which is also exploited in crops (Truniger and Aranda, 2009; Wang and Krishnaswamy, 2012). Recessive resistance traits can be introduced into crop species by crossing, or by random mutagenesis and selection (Piron et al., 2010). Most recessive virus resistance genes isolated to date are the eukaryotic translation initiation factors 4E and 4G, and their isoforms (Hashimoto et al., 2016). Although there has been some progress in the study of soybean resistance to SMV, specific resistance genes and molecular mechanisms of the soybean defense signaling remain largely unknown.

To survive and prevent the invasion and proliferation of pathogens (nematodes, fungi, bacteria, and viruses), plants have developed a complex defense strategy that includes structural (such as cuticular wax, and xylogen deposition on the cell walls) (Kaur et al., 2022) and chemical barriers [such as phenols, saponins, GSLs (glucosinolates), and phytoalexins, etc.] (Zaynab et al., 2018). Once these defenses are breached, host plants immediately activate the PAMPs (pathogen-associated molecular patterns)-triggered immunity (PTI) system. PRRs (pattern recognition receptors) produced by plants can recognize and bind to conserved sequences in microbes, and inhibit pathogen growth (Jones and Dangl, 2006). In addition, effectors secreted by pathogens can activate ETI (effector-triggered immunity) (Howden and Huitema, 2012), resulting in HR (hypersensitive response) and expression of defense-related genes (Win et al., 2012). The transcriptional dynamics of *R* genes and hormone signaling induced by PTI and ETI are crucial for the defense response of plants against the pathogens (Moore et al., 2011).

In recent years, the development of high-throughput sequencing, and other “-omics” studies are providing new insights into the molecular mechanisms of plant defense against viruses and other pathogens (Chen et al., 2017; Awika et al., 2019; Liu et al., 2022). In soybean, the genes involved in SA (salicylic acid) signaling and members of the NBS-LRR (nucleotide binding site leucine-rich repeat) family were involved in SMV pathogenicity (Zhao et al., 2018). Comparative transcriptome analysis of soybean in response to two SMV strains (avirulent strain G5H and virulent strain G7H) revealed that the JA (jasmonic acid) pathway and WRKY transcription factors (TFs) need to be added after these two words. were associated with SMV infection (Alazem et al., 2018). RNA-seq analysis was also used to assess DEGs in soybean under normal and shaded light conditions to investigate the light-regulated response to SMV infection (Zhang L. et al., 2019). Taken together, these studies discussed the effects of hormones, viral strains, and environmental factors (light intensity and quality) on soybean response to SMV, respectively.

The response mechanism of plants to biotic stresses is highly complicated and requires the activation of several combined pathways. Previous studies indicated that the genes associated with cell wall modification, chitinase synthesis, Ca^2+^ signaling, and reactive oxygen gene activation were all significantly up-regulated in pathogen-resistant plants (Zou et al., 2021; Duan et al., 2022; Yang et al., 2022; Zhou et al., 2023). In soybean, the transcriptomic changes during SMV infection are not fully understood. Here, we used two soybean cultivars with different responses to SMV to investigate the physiological and transcriptional changes following SMV infection. Compared to previous studies, we aim to provide more comprehensive information for elucidating the complex regulatory networks associated with the soybean response to SMV. Meanwhile, it is of great theoretical and practical importance to improve disease resistance to SMV through molecular breeding or genetic engineering.

## 2 Materials and methods

### 2.1 Plant materials and virus inoculation

Two breeding lines of *G. max*, ‘Kefeng 1’ (resistant, R-line) and ‘NN1138-2’ (susceptible, S-line) were selected and grown in a greenhouse at 23–28°C with a 14-h light/10-h dark photoperiod. The SMV isolate 6067–1 (strain SC15, GenBank accession: JF833015.1) used in this study was previously collected and sequenced. Leaves of three-leaf stage seedlings were brushed with SMV buffer [prepared by grinding frozen SMV-infected soybean leaves with 600 mesh silicon carbide and 10 mM phosphate buffer (a mixture of NaH_2_PO_4_ and K_2_HPO_4_, pH = 7.4)]. An identical buffer without SMV inoculum was used concurrently for mock treatments. SMV-infected or mock-treated soybean leaves at 1 day post inoculation (dpi) were used as controls for the corresponding materials obtained at subsequent sampling points. Leaf samples taken at 1, 3, 5, 7, 9, and 11 dpi were used to determine SMV accumulation between the two soybean cultivars (three biological replicates per group). All samples were collected, immediately frozen in liquid nitrogen, and stored at −80°C prior to RNA extraction and further analysis.

### 2.2 RNA extraction, cDNA library construction, and Illumina sequencing

Total RNA was extracted using RNAprep Pure Plant Kit (Tiangen, Beijing, China) following the manufacturer’s instructions. The extracted RNA was assessed by 1% agarose gel electrophoresis for degradation, K5800 micro-spectrophotometer (Kaiao, Beijing, China) for concentration and purity, and Bioanalyzer 2100 system (Agilent Technologies, Santa Clara, CA, United States) for integrity. A total of 2 μg RNA per sample was used for cDNA library construction. Sequencing libraries were generated using NEBNext® Ultra™ RNA Library Prep Kit for Illumina® (NEB, Ipswich, MA, United States) following the manufacturer’s instructions. The 48 libraries (RNA samples at 1, 3, 5, and 7 dpi in two cultivars, mock inoculation and virus inoculation, and three biological replicates per group) were sequenced on the Illumina Genome Analyzer IIx at Novogene Bioinformatics Technology Co., Ltd. (Beijing, China).

### 2.3 Bioinformatics analysis

The raw data were filtered by removing the adaptor sequences, N-sequences, and low-quality reads with a quality score Q less than 20. The remaining reads were termed clean reads, and were assembled *de novo* using Trinity software (v2.0.6) with the k-mer size parameter set to 25 by default (Grabherr et al., 2011) to construct full-length transcripts. The longest transcript of each cluster was referred to as a unigene (Wang et al., 2013). The clean reads from each sample were aligned to the *G. max* reference genome^1^ (Schmutz et al., 2010). All unigenes were annotated by NR (NCBI non-redundant protein), Pfam (Protein family), GO (Gene Ontology), KEGG (Kyoto Encyclopedia of Genes and Genomes), and Swiss-Prot protein databases with *e*-value ≤10^−5^.

Gene expression levels were calculated by the FPKM (fragments per kilobase of transcript per million reads) method (Trapnell et al., 2010). The DEGs were analyzed using the DESeq2 R package (v1.18.0) (Love et al., 2014) and defined as unigenes with |log_2_ FC (fold changes)| ≥1, and adjusted *p*-values (padj) < 0.05. GO and KEGG enrichment analyzes were conducted to identify the biological functions of the DEGs using the GOseq R software package (Young et al., 2010) and DAVID Bioinformatics Resources^2^ (Sherman et al., 2022), respectively. Plant Transcription Factor Database^3^ (Jin et al., 2017) was used to identify TFs in all gene sequences. *R* genes were identified using all transcriptome data via the HMM search module in TBtools software (v1.33206.0.0) (Chen et al., 2020). To establish a protein interaction network, candidate proteins in *G. max* were used and analyzed by employing the online STRING database^4^ (Szklarczyk et al., 2021). The interaction network was visualized using Cytoscape 3.7.1 (Shannon et al., 2003), and the betweenness centrality (BC) value of each node was calculated using the CytoNCA plug-in (Tang et al., 2015) in Cytoscape, and was used to show the size of nodes, thus indicating the strength of interactions.

### 2.4 Hormone content measurement

SMV-infected leaf samples were collected using the same method as described for RNA-seq analysis. In addition, samples were collected from non-inoculated and mock-inoculated plants. The extractions were performed according to the protocol (Pan et al., 2010) with minor modifications. The concentrations of endogenous ABA (abscisic acid), CTK (cytokinin, represented by zeatin in this study), IAA (indole-3-acetic acid, a member of the auxins), and SA were quantified using a G6420A HPLC-MS (high-performance liquid chromatography-mass spectrometry) system (Agilent Technologies). The phytohormone standards were purchased from Yuanye (Shanghai, China), and other reagents were purchased from CSP Co., Ltd. (Changshu, China). Each hormone was analyzed in three biological replicates.

### 2.5 qRT-PCR analysis

qRT-PCR (quantitative reverse transcription PCR) assays were performed to confirm the expression of SMV resistance-related genes and to test for the presence or absence of SMV for each soybean line. Reverse transcription was performed with HiScript III 1st Strand cDNA Synthesis Kit (Vazyme, Nanjing, China) following the manufacturer’s instructions. qRT-PCR was performed with ChamQ SYBR qPCR Master Mix (Vazyme) on a LightCycler® 480 II system (Roche, Basel, Switzerland). The expression of each gene was calculated after being normalized to the soybean *GmElF1β* (*elongation factor 1β*) gene (Chen et al., 2015). The relative gene expression level was calculated using the 2^-ΔΔCt^ method. The parameters of a thermal cycle were 95°C for 30 s, followed by 40 cycles of 95°C for 10 s, 60°C for 30 s at a volume of 20 μL, and melting curve analysis. All qPCR experiments were performed in triplicate and primers for qPCR were designed on the Primer-BLAST tool^5^ (Ye et al., 2012) and listed in Supplementary Table S1.

## 3 Results

### 3.1 Phenotype characteristics of inoculated soybean

**Figure 1:**
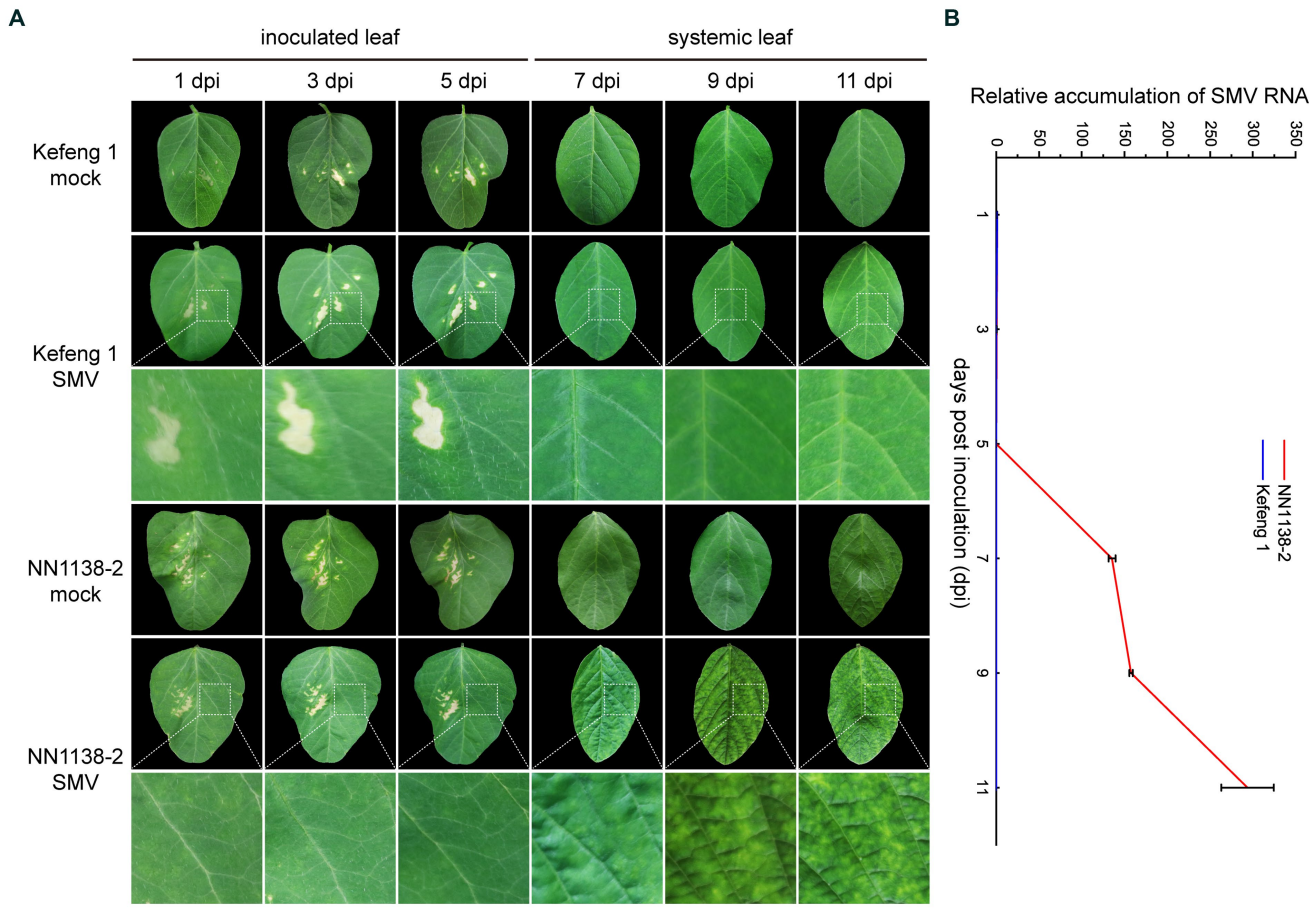
Symptom development and SMV detection in two soybean cultivars. **(A)** Evaluation of the response of ‘NN1138-2’ and ‘Kefeng 1’ to SMV infection in the fully expanded leaf at the top of each plant. Lesions on inoculated leaves from 1 dpi to 5 dpi are caused by brush friction. **(B)** Relative accumulation of SMV RNA (detected by qRT-PCR results of *CP* gene) in inoculated leaves (from 1 dpi to 5 dpi) or systemically infected leaves (from 7 dpi to 11 dpi) of ‘NN1138-2’ and ‘Kefeng 1’ plants, respectively.

After SMV inoculation, no obvious symptoms were observed in either R-line ‘Kefeng 1’ or S-line ‘NN1138-2’ until 5 dpi. S-line shoots showed clear symptoms: wrinkling on infected trifoliate leaves at 7 dpi, chlorosis at 9 dpi, and leaf curling at 11 dpi, whereas all R-line shoots were asymptomatic until 9 dpi and some of them had small yellow spots on leaves at 11 dpi (Figure 1A).

To quantify the relative SMV accumulation in both ‘Kefeng 1’ and ‘NN1138-2’ seedlings, a qRT-PCR assay was performed, using mock-infected seedlings as controls. Virus accumulation was low in both lines from 1 to 5 dpi (compared with 1 dpi, the fold changes of *CP* gene were less than 0.5 in ‘NN1138-2’ and less than 0.1 in ‘Kefeng 1’ from 3 to 5 dpi). Significant differences were detected since 7 dpi, the accumulation of SMV genomic RNA in ‘Kefeng 1’ remained low until the end of the assay (less than 1-fold), while it continued to increase from 9 dpi (more than 157-fold), and reached maximum level at 11 dpi (more than 293-fold) in ‘NN1138-2’ (Figure 1B).

### 3.2 Overview of transcriptome profiles in soybean following SMV infection

After observing the phenotype of soybean inoculated with SMV and calculating the relative expression levels of the *CP* gene (which encodes the viral coat protein), we consider 7 dpi as the onset of the difference in response to SMV between the two soybean cultivars. Therefore, samples at 1, 3, 5, and 7 dpi were selected for transcriptome sequencing. 54,536 genes were obtained after *de novo* assembly (Supplementary Table S2). After filtering and trimming, a total of 2,259,866,196 clean reads were generated from 48 libraries by 150-bp paired-end RNA sequencing (Supplementary Table S3). The quality of the clean data was evaluated by FastQC (Brown et al., 2017). On average, 95.75% of clean reads were mapped to the soybean reference genome [Williams 82 Assembly 4 Annotation 1 (Wm82.a4.v1)] using HISAT2 (Kim et al., 2015), indicating good RNA-seq quality in the present study. According to the total mapped reads, 36,260 genes with an average FPKM of >1 in at least one treatment were considered as expressed genes and further analyzed (Supplementary Figure S1A). Unigenes annotated in NR, Pfam, GO, KO (KEGG ortholog), and Swiss-Prot were used to draw a Venn diagram (Supplementary Figure S1B; Supplementary Table S4), and 7,818 (21.56%) of the unigenes were annotated in these five databases.

**Figure 2:**
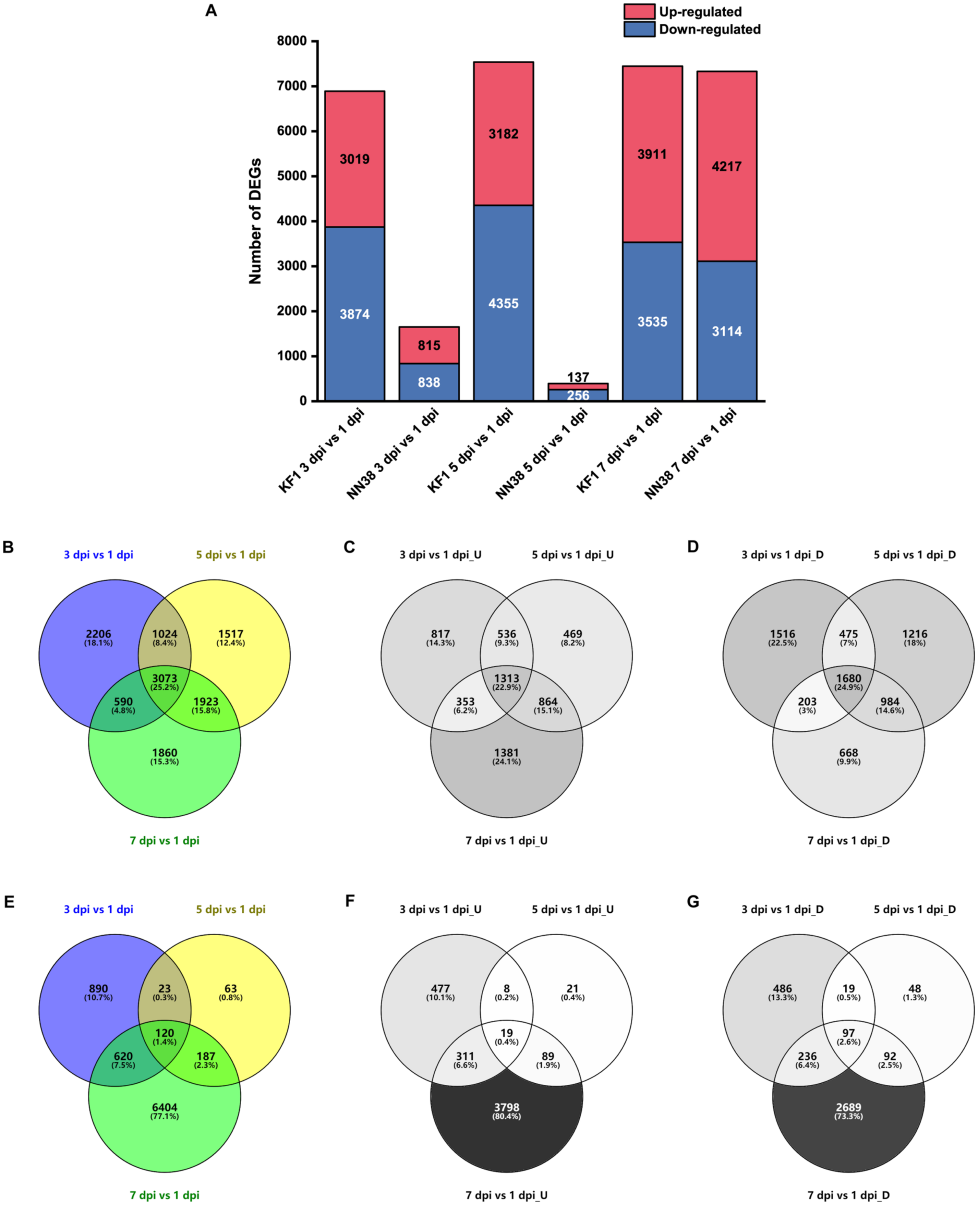
Number of DEGs in SMV-infected soybean seedlings at 3, 5, and 7 dpi compared to 1 dpi. **(A)** Number of up-regulated and down-regulated DEGs at 3, 5, and 7 dpi in ‘Kefeng 1’ (KF1) and ‘NN1138-2’ (NN38). **(B–D)** Venn diagram of total, up-regulated and down-regulated DEGs in ‘Kefeng 1’, respectively. **(E–G)** Venn diagram of total, up-regulated and down-regulated DEGs in ‘NN1138-2’, respectively. DEGs, differentially expressed genes (cutoff ratio of >2, *p*-value <0.05, and *q*-value <0.05). U, up-regulated DEGs; D, down-regulated DEGs.

To evaluate the alterations in unigene expression of SMV-infected soybean, 16,433 DEGs were determined compared with 1 dpi in total, of which 6,893, 7,537, and 7,446 DEGs were identified at 3, 5, and 7 dpi in ‘Kefeng 1’, respectively; and 1,653, 393, and 7,331 DEGs were identified at 3, 5, and 7 dpi in ‘NN1138-2’, respectively (Figure 2A). Furthermore, 5,733 genes were up-regulated at least once, 6,742 genes were down-regulated at least once in three sampling points in ‘Kefeng 1’, and 3,073 DEGs showed a differential expression at all three sampling points, of which 1,313 were upregulated and 1,680 were downregulated (Figures 2B–D). The number of corresponding DEGs in ‘NN1138-2’ at all three sampling points was smaller than that in ‘Kefeng 1’ (Figures 2E–G), and the number of DEGs at 7 dpi of ‘NN1138-2’ is greatly large compared to other time points, indicating that 7 dpi is the initial point for ‘NN1138-2’ to activate the defense response to SMV, which is slower than that of ‘Kefeng 1’.

### 3.3 Contrasting patterns of DEGs between R- and S-lines at the same time point

To investigate the key determinant genes of soybean associated with resistant or susceptible response to SMV, DEGs with opposite expression patterns were analyzed in ‘Kefeng 1’ (R-line) and ‘NN1138-2’ (S-line), respectively. The up-regulated genes in ‘Kefeng 1’ and the down-regulated genes in ‘NN1138-2’ were taken as resistance-response DEGs, while the down-regulated genes in ‘Kefeng 1’ and the up-regulated genes in ‘NN1138-2’ were considered as susceptibility-response DEGs. Similar classification methods were also applied to study the transcriptome dynamics in *Brassica rapa* clubroot caused by *Plasmodiophora brassicae* (Wei et al., 2021) and peanut wilt infected by *Ralstonia solanacearum* (Yang et al., 2022).

At 3 dpi, 53 genes were significantly up-regulated in R-line and down-regulated in S-line. On the contrary, 144 genes were down-regulated in R-line and up-regulated in S-line (Supplementary Figure S2A). At 5 dpi, 5 genes were up-regulated in R-line and down-regulated in S-line, 6 genes were down-regulated in R-line and up-regulated in S-line (Supplementary Figure S2B). At 7 dpi, 47 genes were up-regulated in R-line and down-regulated in S-line, 62 genes were down-regulated in R-line and up-regulated in S-line (Supplementary Figure S2C). These genes are good candidates for functional studies or host breeding for virus resistance, and their possible functions are listed in Supplementary Table S5.

### 3.4 The transcription factors and *R* genes involved in soybean defense response to SMV

Since TFs and *R* genes are important players in plant defense against pathogen infection, we focused on these two types of genes in the study. 3,771 TF genes generated from the Plant Transcription Factor Database and 729 *R* genes predicted by HMMER were annotated (Supplementary Table S6, Sheet 1 and Sheet 2). A total of 1,363 genes encoding TFs and 293 putative *R* genes were differentially expressed once at least at three groups (3 dpi vs. 1 dpi, 5 dpi vs. 1 dpi, and 7 dpi vs. 1 dpi) of both cultivars inoculated with SMV (Supplementary Table S6, Sheet 3 and Sheet 4).

**Figure 3:**
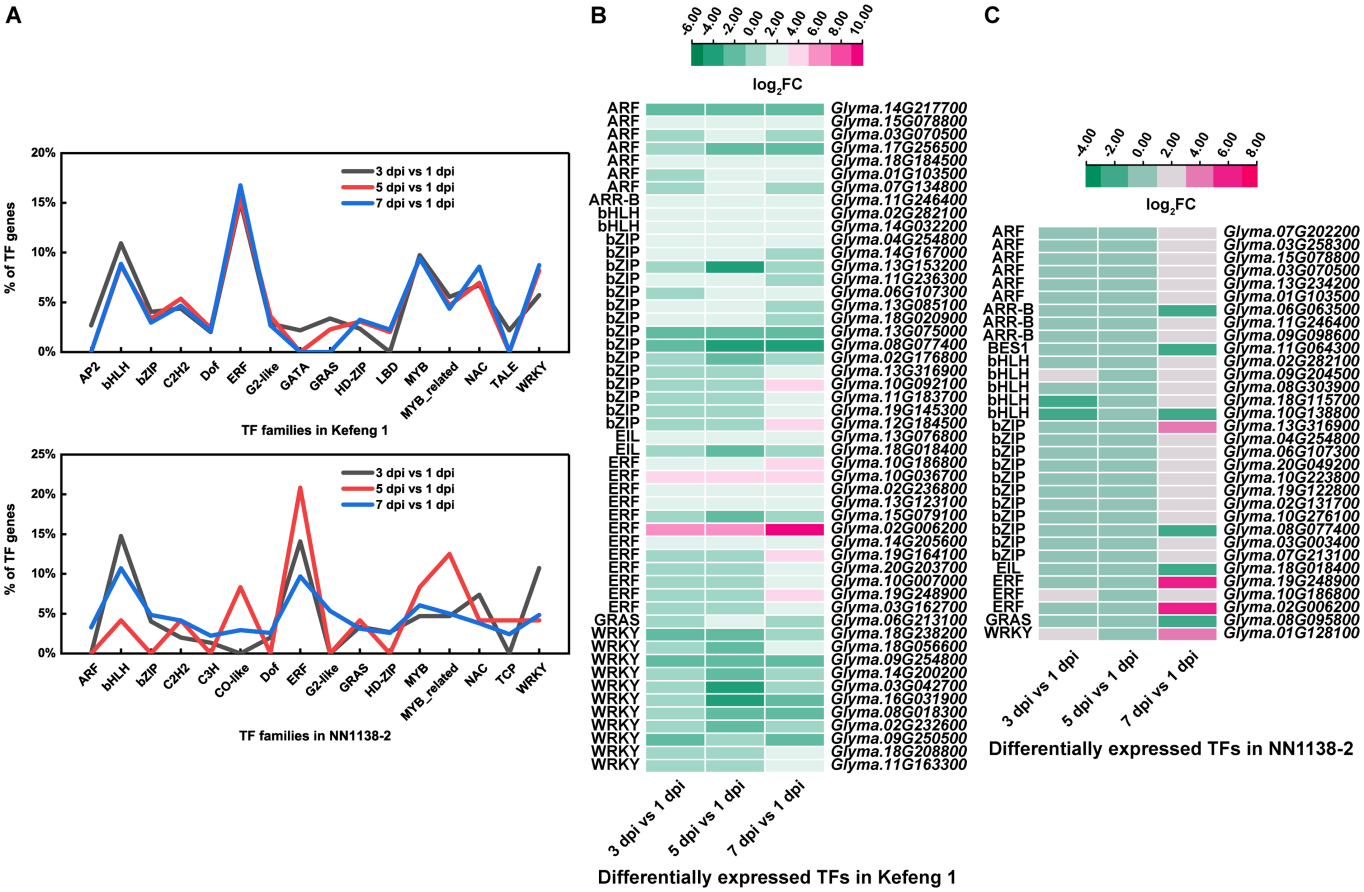
Differentially expressed transcription factors (TFs) in two soybean cultivars. **(A)** The percentage of TFs among all DEGs in SMV-infected soybean seedlings at 3, 5, and 7 dpi coupled with 1 dpi. **(B)** Heatmap of differentially expressed TFs enriched in three KEGG pathways in ‘Kefeng 1’. **(C)** Heatmap of differentially expressed TFs enriched in three KEGG pathways in ‘NN1138-2’. The expression level was calculated using log_2_(FC). FC, fold change.

The number of differentially expressed TF genes in each group was listed in Table 1. TF families with larger number of DEGs were shown in Figure 3A. In ‘Kefeng 1’, the majority of differentially expressed TFs belong to the ERF family, followed by MYB, bHLH, and WRKY families. In addition, the bHLH family has the most DEGs in ‘NN1138-2’, followed by ERF, WRKY, and MYB families. Since a large number of TF genes were differentially expressed in soybean after SMV infection, we subsequently focused on those genes enriched in plant hormone signal transduction, plant-pathogen interaction, and MAPK (mitogen-activated protein kinase) signaling pathways, because these three metabolic pathways have been reported to play important roles in the process of pathogen infection in host plants. There were 51 TF genes enriched in the above three KEGG pathways in ‘Kefeng 1’, and the heatmap showed that 2/3 of them were up-regulated after SMV inoculation (Figure 3B). In ‘NN1138-2’, none of the TFs were enriched in MAPK signaling pathway and the DEGs at 5 dpi were not enriched in any of the pathways. A total of 32 TFs were enriched in plant hormone signal transduction and plant-pathogen interaction pathways, and 26 TFs showed upregulated expression (Figure 3C).

**Table 1:** The number of differentially expressed TF genes in each group.

Comparison group	Up_TF	Down_TF	Total
R3 vs R1	303	281	584
R5 vs R1	343	392	735
R7 vs R1	413	289	702
S3 vs S1	94	55	149
S5 vs S1	10	14	24
S7 vs S1	339	234	573

Up_TF and Down_TF indicate up-regulated and down-regulated TFs, respectively. R1, R3, R5, and R7 represent the resistant soybean leaves inoculated with SMV at 1, 3, 5, and 7 dpi, respectively; S1, S3, S5, and S7 indicate the susceptible soybean leaves inoculated with SMV at 1, 3, 5, and 7 dpi, respectively.

**Figure 4:**
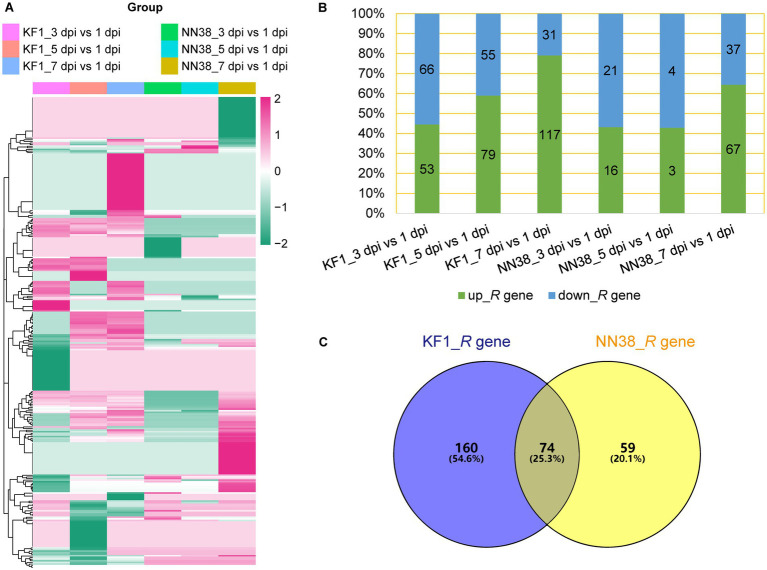
Differential expression of putative *R* genes in two soybean cultivars. **(A)** Heatmap showing the expression profile of putative *R* genes upon SMV infection. **(B)** Number and percentage of up- and down-regulated *R* genes in each group. **(C)** Venn diagram showing individual and common differentially expressed *R* genes. KF1: ‘Kefeng 1’ (resistant line); NN38: ‘NN1138-2’ (susceptible line).

Two hundred thirty-four putative *R* genes were differentially expressed in ‘Kefeng 1’, and 106 of them were down-regulated in at least one group (Figure 4A; Supplementary Table S6, Sheet 4), 66 genes were down-regulated in the 3 dpi vs. 1 dpi group, which was the highest number and percentage among the three groups (Figure 4B). Moreover, 133 putative *R* genes were differentially expressed in ‘NN1138-2’, which was significantly lower than the number in ‘Kefeng 1’, and 56 of them were down-regulated in at least one group, which was slightly lower than the proportion of corresponding genes in ‘Kefeng 1’ (Figure 4A; Supplementary Table S6, Sheet 4). Interestingly, when the down-regulated *R* genes in three groups of NN118-2 were compared, among which 37 genes were found in the 7 dpi vs. 1 dpi group, which was the largest number but the smallest proportion among the three groups (Figure 4B). The Venn diagram also showed that 74 common *R* genes were differentially expressed in both cultivars (Figure 4C).

### 3.5 Functional enrichment analyzes of DEGs

To investigate the key biological progresses and pathways involved in soybean defense against SMV, all the up-regulated and down-regulated DEGs were annotated in GO database, separately. For example, up-regulated DEGs in ‘Kefeng 1’ can be enriched in plant-type secondary cell wall biogenesis (GO:0009834), and down-regulated DEGs in both cultivars can be enriched in cell wall (GO:0005618), cell wall biogenesis (GO:0042546) and cell wall organization (GO:0071555; Supplementary Figure S3). Detailed information on these three groups of DEGs is provided in Supplementary Table S7.

**Figure 5:**
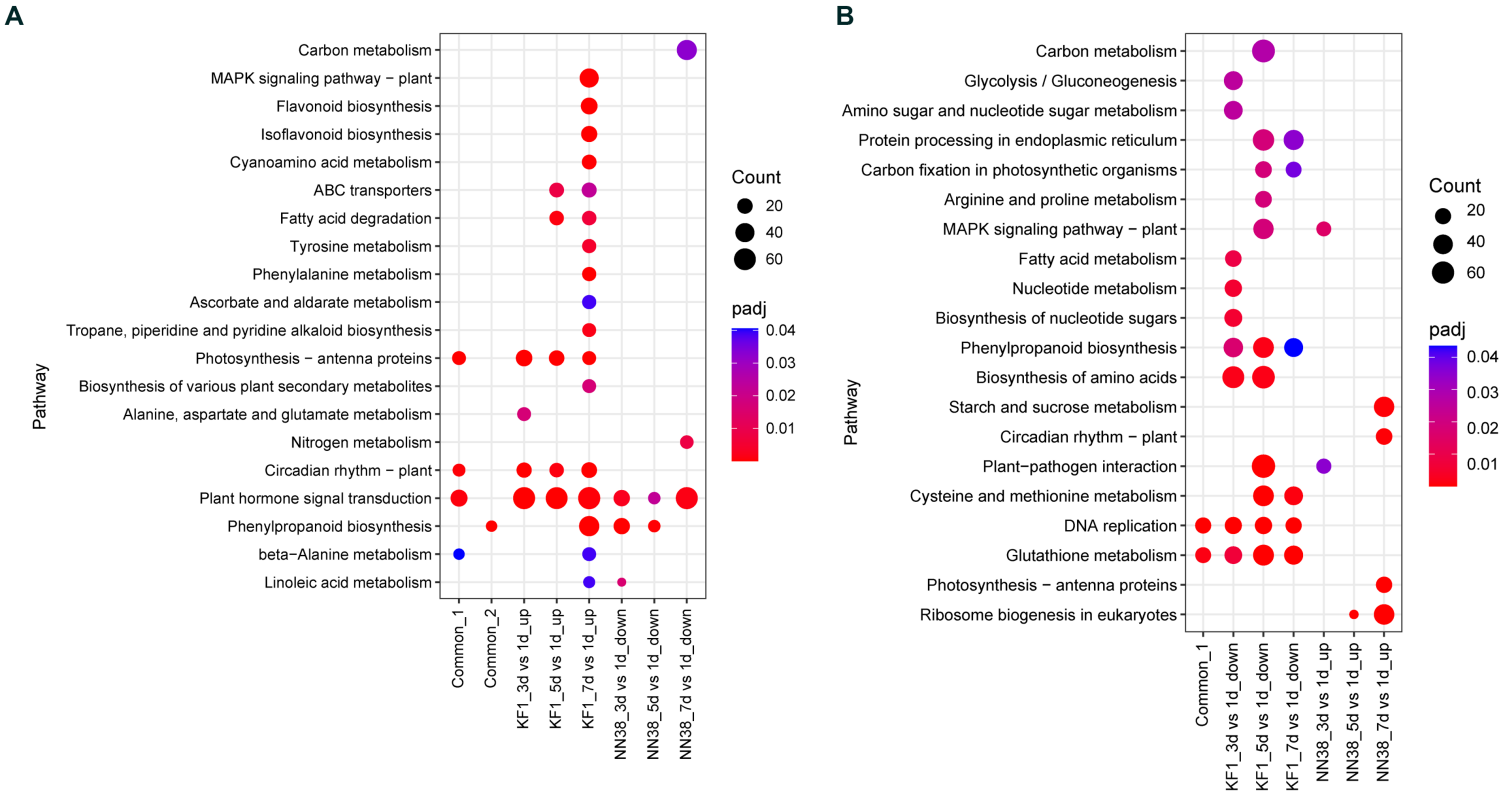
KEGG enrichment analysis of common, up-regulated, and down-regulated DEGs in each treatment comparison in ‘Kefeng 1’ (KF1) and ‘NN1138-2’ (NN38) seedlings, respectively. **(A)** Common_1 indicates the common up-regulated DEGs in KF1_3 dpi vs. 1 dpi, KF1_5 dpi vs. 1 dpi, and KF1_7 dpi vs. 1 dpi. Common_2 indicates the common down-regulated DEGs in NN38_3 dpi vs. 1 dpi, NN38_5 dpi vs. 1 dpi, and NN38_7 dpi vs. 1 dpi. **(B)** Common_1 indicates the common down-regulated DEGs in KF1_3 dpi vs. 1 dpi, KF1_5 dpi vs. 1 dpi, and KF1_7 dpi vs. 1 dpi. No common_2 in the *x*-axis because the common up-regulated DEGs in NN38_3 dpi vs. 1 dpi, NN38_5 dpi vs. 1 dpi, and NN38_7 dpi vs. 1 dpi cannot be enriched in any KEGG pathway with padj <0.05.

These up-regulated and down-regulated DEGs in both cultivars were also used for KEGG enrichment analysis, respectively. Resistance-response DEGs and susceptibility-response DEGs were performed to investigate the biological mechanisms of interaction between soybean and SMV. The significantly enriched KEGG pathways are summarized in Figure 5, and the genes under each pathway are listed in Supplementary Table S8.

#### 3.5.1 Resistance-response DEGs

When the leaves of ‘Kefeng 1’ seedlings were infected with SMV, a number of genes were induced. It is widely believed that these genes are involved in the activation of immune responses. There are 3,019, 3,182, and 3,911 up-regulated DEGs in KF1_3 dpi vs. KF1_1 dpi, KF1_5 dpi vs. KF1_1 dpi, and KF1_7 dpi vs. KF1_1 dpi, respectively (Figure 2A). KEGG enrichment analysis showed that the number of DEGs enriched in plant hormone signal transduction (gmx04075), photosynthesis-antenna proteins (gmx00196), circadian rhythm-plant (gmx04712) and MAPK signaling pathway-plant (gmx04016) ranked the top four among the up-regulated DEGs of ‘Kefeng 1’ (Figure 5A; Supplementary Table S8, Sheet 1).

Similarly, the expression of many genes was reduced in the leaves of susceptible soybean ‘NN1138-2’ when infected with SMV. These genes were also resistance response genes because they negatively regulated susceptibility. There are 838, 256, and 3,114 down-regulated DEGs in NN38_3 dpi vs. NN38_1 dpi, NN38_5 dpi vs. NN38_1 dpi, and NN38_7 dpi vs. NN38_1 dpi, respectively (Figure 2A). The number of significantly enriched KEGG pathways involved in the down-regulated DEGs of ‘NN1138-2’ is much smaller than those involved in the up-regulated DEGs of ‘Kefeng 1’ (Figure 5A).

To further narrow down the scope of vital candidate resistance-response genes, three groups of DEGs in ‘Kefeng 1’ and ‘NN1138-2’ were subjected to Venn analysis to find the common DEGs, and 1,313 DEGs in ‘Kefeng 1’ and 97 DEGs in ‘NN1138-2’ were obtained, respectively (Figures 2C,G). KEGG enrichment analysis suggested that the common DEGs in ‘Kefeng 1’ were enriched in photosynthesis-antenna proteins, circadian rhythm-plant, plant hormone signal transduction, and β-Alanine metabolism (gmx00410), and the common DEGs in ‘NN1138-2’ were enriched only in phenylpropanoid biosynthesis (Figure 5A).

#### 3.5.2 Susceptibility-response DEGs

As SMV enters into host cells, the virulence factors will attack the plant’s immune system. The expression of several genes will be increased in susceptible genotypes. To investigate these susceptibility-response DEGs, the transcriptome dynamics of SMV-infected ‘NN1138-2’ leaves were examined. There are 815, 137, and 4,217 up-regulated DEGs in NN38_3 dpi vs. NN38_1 dpi, NN38_5 dpi vs. NN38_1 dpi, and NN38_7 dpi vs. NN38_1 dpi, respectively (Figure 2A). The number of up-regulated DEGs in NN38_7 dpi vs. NN38_1 dpi was significantly higher than those in the other two comparison groups, and DEGs in this group were involved in starch and sucrose metabolism (gmx00500), circadian rhythm-plant, photosynthesis-antenna proteins, and ribosome biogenesis in eukaryotes (gmx03008). Several upregulated DEGs in NN38_3 dpi vs. NN38_1 dpi were enriched in MAPK signaling pathway-plant and plant-pathogen interaction (gmx04626). Ribosome biogenesis in eukaryotes was the significant enrichment KEGG pathway associated with up-regulated DEGs of NN38_5 dpi vs. NN38_1 dpi (Figure 5B). Venn analysis revealed that 19 DEGs were common in these comparison groups (Figure 2F), but these DEGs cannot be enriched in any of the KEGG pathways due to their small account.

Furthermore, when the ‘Kefeng 1’ plants were infected with SMV, the expression of some genes was decreased. These genes were also considered as susceptibility-response genes. There were 3,874, 4,355, and 3,535 down-regulated DEGs in KF1_3 dpi vs. KF1_1 dpi, KF1_5 dpi vs. KF1_1 dpi, and KF1_7 dpi vs. KF1_1 dpi, respectively (Figure 2A). Biosynthesis of amino acids (gmx01230), protein processing in endoplasmic reticulum (gmx04141), phenylpropanoid biosynthesis, and cysteine and methionine metabolism (gmx00270) were the top 4 significantly enriched KEGG pathways among these down-regulated DEGs. 1,680 common DEGs were identified by Venn analysis (Figure 2D), and these genes were significantly enriched in DNA replication and glutathione metabolism pathways (Figure 5B; Supplementary Table S8, Sheet 2).

### 3.6 Plant hormone signal transduction contributed to defense response

**Figure 6:**
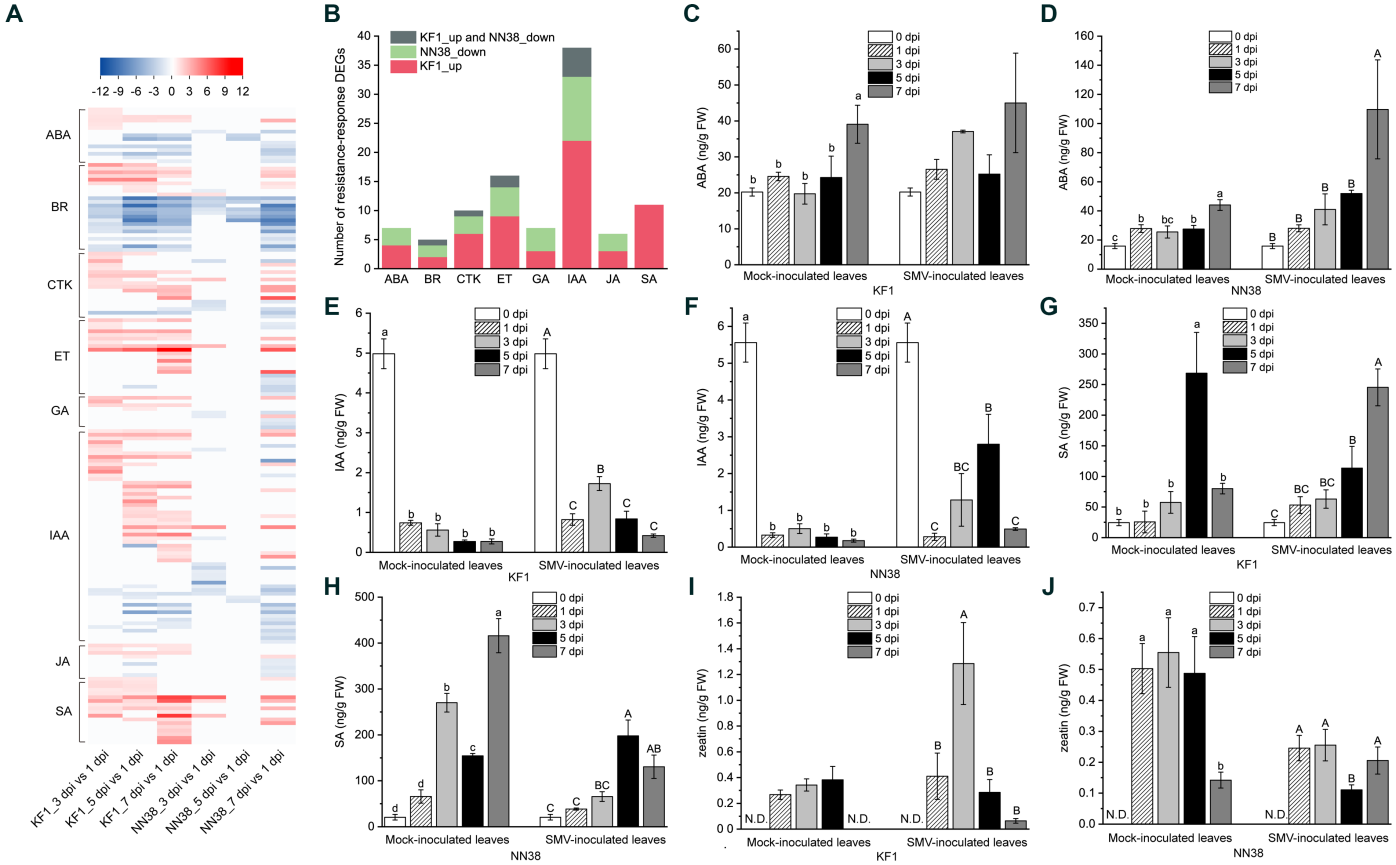
Expression analysis of phytohormone-related DEGs and quantitative analysis of four representative hormones in soybean leaves. **(A)** Heatmap analysis of phytohormone-related DEGs. **(B)** Number of resistance-response DEGs associated with each hormone. ABA **(C,D)**, IAA **(E,F)**, SA **(G,H)**, and CTK (zeatin) **(I,J)** contents in uninfected (mock-inoculated) and infected (SMV-inoculated) leaves of Kefeng 1 (KF1) and NN1138-2 (NN38) plants. Values are the mean ± SE (standard error) of three biological replicates per treatment. Different letters above each column indicate a significant difference (upper and lower case letters suggest that the two sets of data were significantly analyzed independently; *p* < 0.05; *n* = 3). FW indicates fresh weight of soybean leaves and N.D. represents that zeatin content was not detectable in this sample.

KEGG enrichment analysis showed that the number of DEGs enriched in the “plant hormone signal transduction” pathway was the highest among all immune response-related pathways, and this pathway was also the unique that could enrich all three groups of DEGs in both cultivars (Figure 5A; Supplementary Table S8, Sheet 1). SMV infection positively or negatively regulated the expression of genes associated with hormone [IAA, ET (ethylene), SA, CTK, ABA, GA (gibberellic acid), JA, and BR (brassinosteroid)] signaling but with quantitative differences in two cultivars (Figure 6A; Supplementary Table S9).

In this study, 58 DEGs were associated with IAA signaling, which was the highest among all hormones. The transcriptome sequencing data clearly showed that 5 dpi was the most important time point for IAA signaling compared to other inoculation time points, as the majority of up-regulated IAA-related genes were in the 5 dpi vs. 1 dpi group (23 genes, e.g., *IAA4-like*, *GH3.6*, *SAUR32*, and *LAX12*), and most of the down-regulated IAA-related genes were also in this group (8 genes, e.g., *GH3.2*, *SAUR71*, and *IAA13-like*). Six DEGs encoding PP2C (protein phosphatase 2C) in the ABA signaling pathway were differentially expressed, only one gene (*Glyma.09G066500*) was up-regulated, while the rest of the genes were down-regulated. Cytokinins have been shown to regulate the expression of defense genes and the activation of immune responses (Choi et al., 2010). Most of the genes related to CTK signaling (13/18) were upregulated in ‘Kefeng 1’, including four *ARR* genes (homologs of *Arabidopsis* response regulator).

After SMV infection, only the DEGs enriched in SA signaling were all up-regulated in both cultivars (Figure 6A; Supplementary Table S9), these DEGs can be divided into three categories: *bZIP* (basic leucine zipper) transcription factors, *PR1* (pathogenesis-related protein 1), and *NPR1* (non-expressor of PR1). The family of TFs containing a bZIP domain is one of the largest families of TFs in plants that can regulate genes in response to seed maturation, flower development, abiotic stress, and pathogen defense (Jakoby et al., 2002). 7 members of the bZIP TFs contain the so-called TGA (TGACG *cis*-DNA binding) motif (they are considered members of the TGA subfamily, see Supplementary Table S9), their homologs in *A. thaliana* act as regulators in SA signaling and are linked to biotic stress responses (Singh et al., 2002; Dröge-Laser et al., 2018). *NPR1* acts as a key regulator of SA-mediated resistance in *A. thaliana*, its two homologs in soybean (*Glyma.14G031300* and *Glyma.02G283300*) were up-regulated in ‘Kefeng 1’, but did not show differential expression in ‘NN1138-2’ compared to the control.

To further explore the relationship between hormone signaling and soybean resistance to SMV, a bar graph of the number of resistance-response DEGs associated with eight hormones was plotted (Figure 6B). The number of DEGs involved in IAA, ET, SA, CTK, and ABA signaling ranked among the top 5 of all hormones. Since ethylene is gaseous under natural conditions, the levels of the remaining four intracellular hormones were measured. The ABA content in SMV-inoculated ‘NN1138-2’ seedlings at 7 dpi was significantly higher than that of the other four sampling points, while there were no significant differences among the five sampling points in ‘Kefeng’ (Figures 6C,D). Compared with non-inoculated leaves, IAA content in inoculated leaves was significantly reduced (*p* < 0.05) during both mock inoculation and SMV infection in two cultivars (Figures 6E,F). In contrast, the SA content of both cultivars was significantly higher in at least one sampling point (mock inoculation or SMV inoculation) than in the uninoculated state (Figures 6G,H). An interesting finding was that the level of zeatin in ‘Kefeng 1’ after SMV infection was higher than that in the mock inoculation group, while the level of this hormone in ‘NN1138-2’ showed the opposite trend (Figures 6I,J).

### 3.7 Starch and sucrose metabolism and Ca^2+^ signaling regulated susceptible response to SMV

Only susceptibility-response DEGs in ‘NN1138-2’ can be enriched in starch and sucrose metabolism (Figure 5B). There are 45 DEGs enriched in this pathway, including nine genes encoding glucose-1-phosphate adenylyltransferase large (or small) subunits (*AGP*, ENZYME entry: EC 2.7.7.27), six genes encoding β-glucosidase (*BGLU*, EC 3.2.1.21), five genes encoding starch synthase (*SS*, EC 2.4.1.242), four genes encoding endoglucanase (*EG*, EC 3.2.1.4), three genes encoding glucose-6-phosphate isomerase (*G6P*, EC 5.3.1.9), three genes encoding sucrose synthase (*SUS*, EC 2.4.1.13), three genes encoding trehalose-6-phosphate phosphatase (*TPP*, EC 3.1.3.12), two genes encoding hexokinase-1 (*HXK1*, EC 2.7.1.1), and other genes that modify the structure of starch or its intermediates (Bolouri Moghaddam and Van den Ende, 2012; Jeandet et al., 2022). However, the majority of these members (25/45) were not differentially expressed in ‘Kefeng 1’ after SMV inoculation (Supplementary Table S10, Sheet 1). The results suggest that genes related to sugar metabolism play an important role in the interaction between SMV and ‘NN1138-2’ rather than ‘Kefeng 1’, and 7 dpi is the starting point of this immune response.

**Figure 7:**
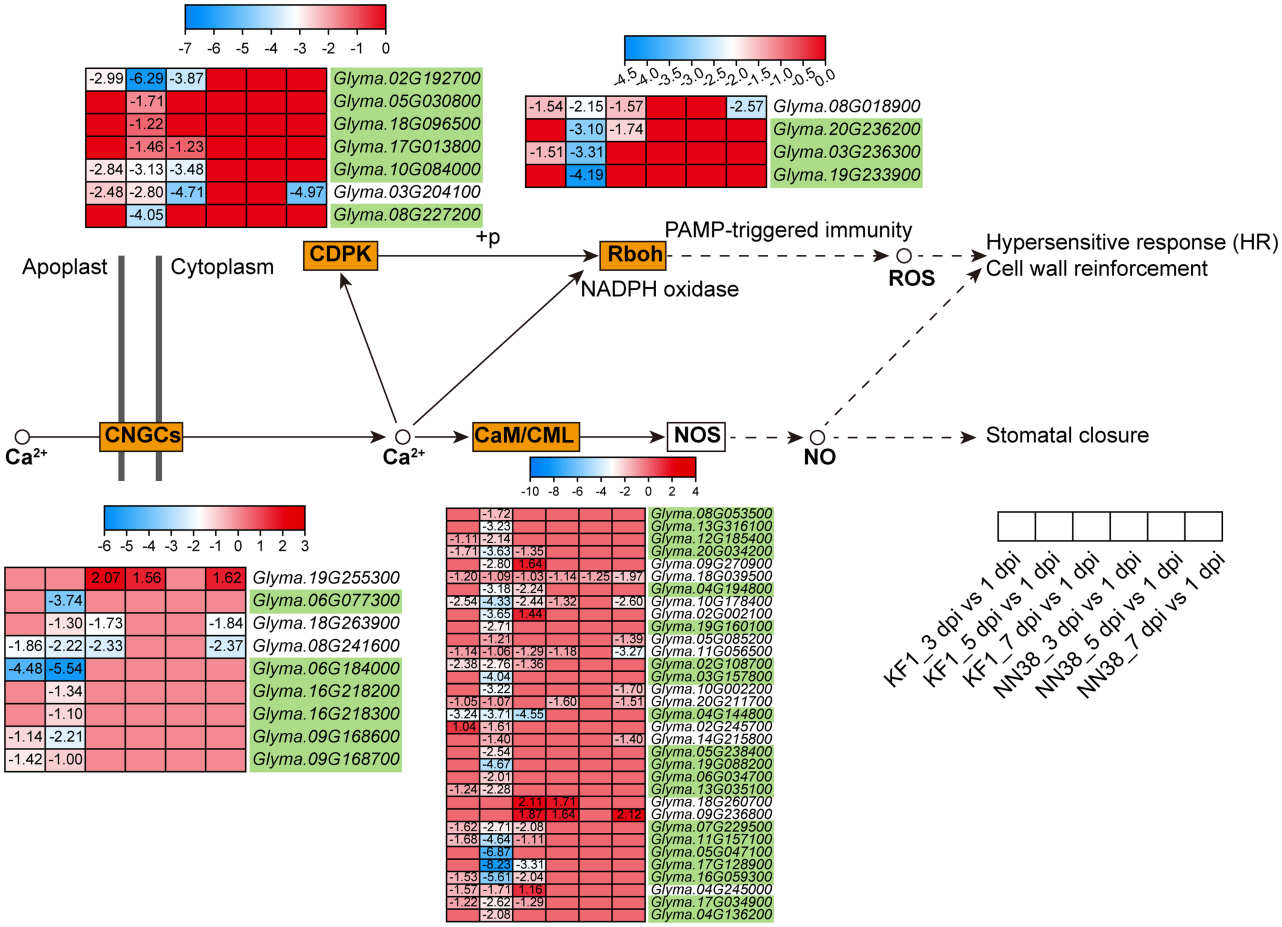
Expression profiles of key DEGs involved in the Ca^2+^ signaling pathway in two soybean cultivars after SMV inoculation. The gene IDs highlighted in yellowgreen indicate the susceptibility-response DEGs. KF1: ‘Kefeng 1’; NN38: ‘NN1138-2’.

Calcium ion (Ca^2+^) is a universal secondary messenger involved in all aspects of life, including growth regulation, development, reproduction, abiotic stresses, and other environmental stimuli (Kudla et al., 2018). In this study, 53 Ca^2+^ signaling-related DEGs were enriched in plant-pathogen interactions (Figure 7; Supplementary Table S10, Sheet 2). These genes were grouped into four subfamilies: CaM (calmodulin) and CMLs (CaM-like proteins), CDPKs (calcium-dependent protein kinases), CNGCs (cyclic nucleotide-gated ion channels), and Rboh (respiratory burst oxidase homolog protein). Notably, when pathogens invade plant cells, cellular responses during both PTI and ETI involve dynamic changes in cytosolic Ca^2+^ concentrations (Zhivotovsky and Orrenius, 2011; Yuan P. et al., 2020; Yuan et al., 2021). Therefore, the differential expression of Ca^2+^ signaling-related genes regulates dynamic changes in cytosolic Ca^2+^ concentrations, which is an early event during immune responses. Compared with other groups, the 5 dpi vs. 1 dpi group in ‘Kefeng 1’ had the largest number of DEGs (50), and 35 Ca^2+^ signaling-related DEGs were susceptibility-response genes as they were downregulated in ‘Kefeng 1’ and not differentially expressed in ‘NN1138-2’.

### 3.8 MAPK signaling pathway regulated both defense and susceptible responses to SMV

Forty DEGs in ‘Kefeng 1’ were enriched in MAPK signaling pathway, and half of them were also enriched in plant hormone signal transduction. Among the 20 DEGs exclusively enriched in MAPK signaling pathway (Supplementary Table S11, Sheet 1), *CHIA1* (chitinase class I, *Glyma.02G042500*), *MAPKK9* (mitogen-activated protein kinase kinase 9, *Glyma.07G105700*) and a member of *MKS1* (*MKS1-1*, a MAP kinase substrate: *Glyma.04G239400*) were up-regulated in at least two sampling points in ‘Kefeng 1’, but they were not differentially expressed in ‘NN1138-2’. The expression patterns of the above three genes in soybean suggest that they are involved in the biosynthesis of defense-related secondary metabolites and play an important role in ‘Kefeng 1’ resistance to SMV. Furthermore, the homolog of RbohA (respiratory burst oxidase homolog protein A, *Glyma.01G222700*) in rice (*Oryza sativa*) triggers the pathogen-induced ROS (reactive oxygen species) burst (Nagano et al., 2016). CML has been linked to cell signaling and a variety of biotic and abiotic stimuli (Zhang et al., 2014; Yuan et al., 2017), and EPF (epidermal patterning factor) has been correlated to regulate many aspects of plant growth and development (Lu et al., 2019). Several homologous genes of these proteins were up-regulated in both ‘Kefeng 1’ and ‘NN1138-2’ but with variant expression levels. These results suggest that partial members of RbohAs, CMLs and EPFs are involved in the regulation of soybean resistance to SMV.

Some candidate susceptibility-related DEGs in both cultivars: down-regulated DEGs in ‘Kefeng 1’ (5 dpi vs. 1 dpi) and up-regulated DEGs in ‘NN1138-2’ (3 dpi vs. 1 dpi) were also enriched in MAPK signaling pathway (Figure 5B). Ten of them were simultaneously enriched in plant hormone signal transduction, were excluded, and the expression profiles of the remaining 50 genes are listed in Supplementary Table S11, Sheet 2. Among them, 46 genes were differentially expressed in ‘‘‘Kefeng 1’ vs. CK” (meaning one of the three groups in ‘Kefeng 1’), of which six were up-regulated, 38 were down-regulated, and two were up- or down-regulated. It is noteworthy that three genes encoding PP2C proteins (*Glyma.01G225100*, *Glyma.11G222600*, and *Glyma.11G018000*) were significantly down-regulated in ‘Kefeng 1’ after SMV infection, while they were not differentially expressed in ‘NN1138-2’, suggesting that they may be involved in the negative regulation of SMV resistance in soybean. A similar report in *A. thaliana* showed that PP2C38 acts as a negative regulator of BIK1 (brassinosteroid-insensitive 1-associated receptor kinase 1) activity and BIK1-mediated immunity (Couto et al., 2016).

Combining the expression patterns of DEGs enriched in the MAPK signaling pathway in two cultivars, 39 genes were susceptibility-response DEGs, of which 35 genes were downregulated in ‘Kefeng 1’ and not differentially expressed in ‘NN1138-2’, and the remaining four genes (another gene encoding MKS1, *MKS1-2*: *Glyma.05G190000*, *WRKY5*: *Glyma.01G128100*, *MAPKK2*: *Glyma.15G172600*, and *MYC1*: *Glyma.09G204500*) were not differentially expressed in ‘Kefeng 1’ but upregulated in ‘NN1138-2’. An interesting finding was that most of the WRKY transcription factors enriched in the MAPK signaling pathway (7/11) were involved in the susceptibility response to SMV, suggesting that these WRKY proteins negatively regulate soybean defense against SMV. Previous studies indicated that WRKYs regulate transcription, signaling, plant defense, and other physiological processes by interacting with a variety of plant proteins (Chi et al., 2013; Wani et al., 2021). However, little is known about the transcriptional regulation of WRKY transcription factors during plant virus infection. Therefore, their regulatory mechanisms in soybean-SMV interaction will be of great significance in the future.

### 3.9 Protein–protein interaction analysis revealed circadian clock associated genes-TFs-MAPKs interactions might be involved in defense response

**Figure 8:**
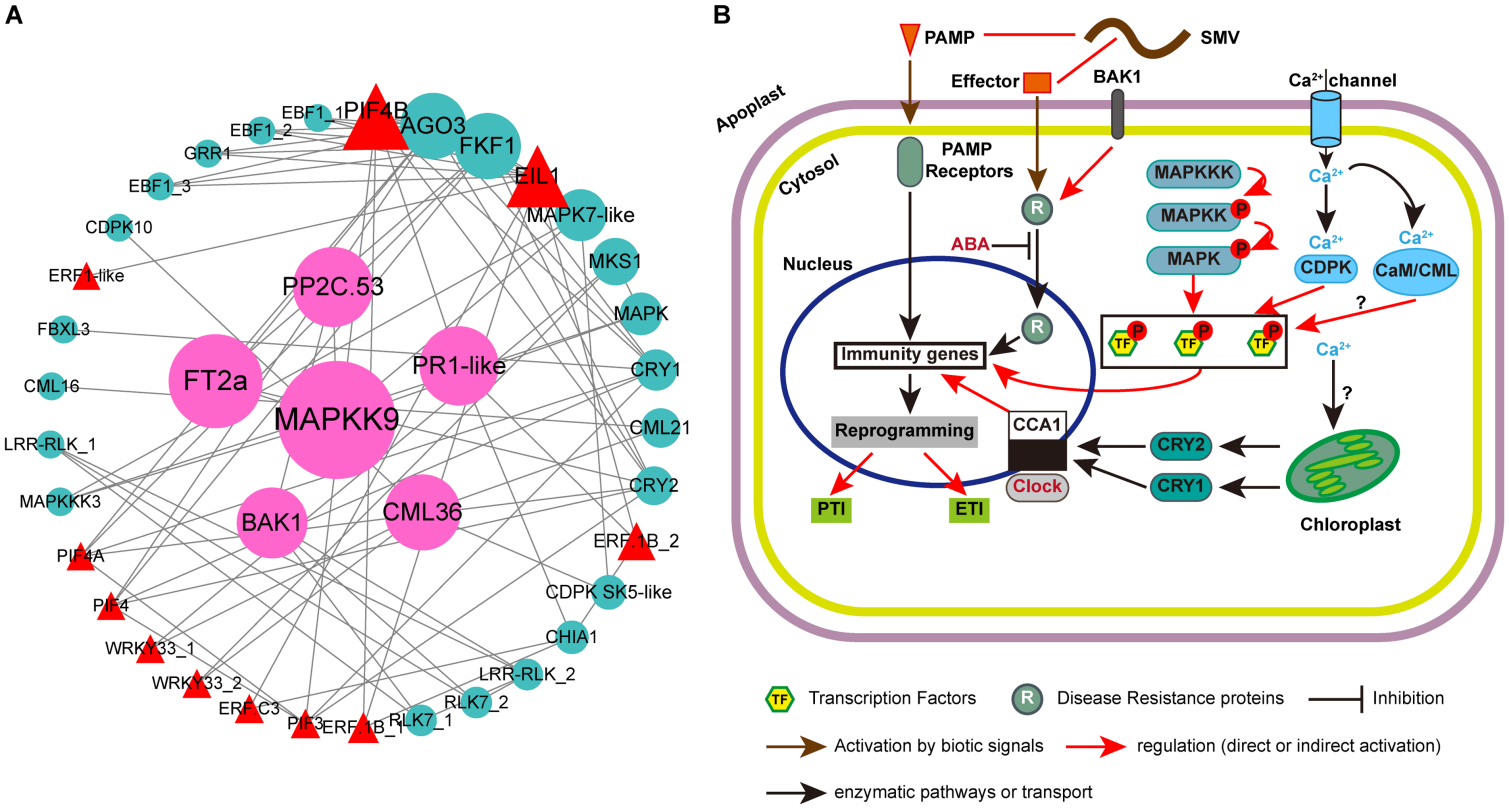
Protein-protein interaction (PPI) analysis of resistance-response DEGs and simple schematic diagram of soybean response to SMV in our study. **(A)** PPI network generated from 39 resistance-response DEGs. Circles represent the nodes of proteins, and the sizes of the circles represent the intensity of protein interactions as inferred by the value of the betweenness parameter. Lines between two nodes indicate that two proteins may interact. Cyan circles represent proteins encoded by DEGs, red triangles represent transcription factors (TFs), and pink circles represent proteins encoded by candidate hub genes. **(B)** Modulation of plant immune responses by MAPK cascades, clock proteins, TFs, and Ca^2+^ signaling. Note that not all components of abiotic and immune responses are depicted and interactions between defense molecules (such as between SA, JA, and ET) or abiotic factors (such as between light and clock) are not illustrated.

Plant immunity is regulated by a complex network of proteins. Previous studies have confirmed the roles of multiple MAPKs, R proteins, TFs and hormone-related proteins in resistance to plant pathogens (Robert-Seilaniantz et al., 2011; Spoel and Dong, 2012; Xu et al., 2018; Tellez et al., 2020; Wani et al., 2021). However, their synergistic regulation of plant responses to viruses has not been reported. In this study, besides plant hormone signal transduction, photosynthesis-antenna proteins and circadian rhythm-plant are two other KEGG pathways that can enrich all the three groups of up-regulated DEGs in ‘Kefeng 1’ (Figure 5A). The DEGs enriched in photosynthesis-antenna proteins or circadian rhythm-plant pathways were submitted to STRING database with the above four types of proteins to construct a protein–protein interaction (PPI) network. The majority of photosynthesis-antenna proteins do not interact with these proteins (data not shown), while the interaction network generated from six circadian rhythm-related proteins and these resistance-response proteins was predicted from 39 node proteins with the enrichment *p* value <1.0 × 10^−16^ at the medium confidence parameter level (Figure 8A; Supplementary Table S12, Sheet 1).

There were 77 combinations (also known as edges) in the PPI network, of which 10 interactions occurred between the clock proteins (proteins enriched in the circadian rhythm-plant pathway; Supplementary Table S12, Sheet 2). A member of CCAs (circadian clock-associated proteins): FKF1 (flavin-binding, kelch repeat, f-box 1: *Glyma.05G239400*) had 12 potential interacting partners, and four members of them [EIN3 (ETHYLENE INSENSITIVE 3)-like 1 (EIL1): *Glyma.13G076800* and three EBF1 (EIN3-binding F-box protein 1) genes: *Glyma.13G166200, Glyma.04G066900* and *Glyma.14G116800*] are involved in the ethylene signaling pathway. Another clock protein: ADO3 (adagio protein 3, *Glyma.08G046500*) may also interact with EIL1 and EBF1. A previous study indicated that the *A. thaliana* ADO3 homolog is a component of an E3 ubiquitin ligase complex that plays a central role in blue light-dependent circadian cycles (Jarillo et al., 2001). Since most of the ethylene signaling-related DEGs (16/21) are resistance-response genes (Supplementary Table S9), we suppose that endogenous ethylene plays an important role in the immune response of soybean to SMV and this process is regulated by FKF1 protein and blue light.

Phytochrome-Interacting Factors (PIFs) are members of the basic helix–loop–helix (bHLH) domain-containing transcription factor superfamily, and were originally recognized for their role in promoting plant growth (Li et al., 2012; Park et al., 2012). Recent studies have demonstrated novel functions of PIFs in regulating multiple signaling pathways: endogenous (e.g., hormonal) as well as abiotic (light, circadian, and elevated temperature) and biotic (defense responses) pathways (Sun et al., 2012; Xu et al., 2015; De Wit et al., 2016; Soy et al., 2016; Zust and Agrawal, 2017; Zhao et al., 2021). Phytochromes and cryptochromes (CRYs) have been reported as photoreceptors responsible for the light entrainment of the circadian clock in *A. thaliana* (Somers et al., 1998). Our PPI prediction suggested that two blue light photoreceptors: CRY1 (*Glyma.06G103200*) and CRY2 (*Glyma.10G180600*) may interact with four PIFs (PIF3, PIF4, PIF4A, and PIF4B) (Figure 8A; Supplementary Table S12, Sheet 2). Studies on the Arabidopsis-*turnip crinkle virus* (TCV) pathosystem revealed that CRY1 and CRY2, together with PHOT1 (phototropin 1, another blue light receptor) and PHOT2, but not phytochromes, are required for resistance to TCV (Chandra-Shekara et al., 2006; Jeong et al., 2010a,b). Prediction also suggests that CRY1 may interact with a candidate R protein: F-box/LRR-repeat protein 3 (FBXL3, *Glyma.14G200300*). All these results demonstrated that the PIF-CRY-R protein module might mediate soybean resistance to SMV.

In addition to EIL1 and PIF transcription factors, two genes (*Glyma.09G280200* and *Glyma.18G208800*) encoding putative GmWRKY33 in the plant-pathogen interaction pathway, could interact with MKS1 (*Glyma.04G239400*; Figure 8A). These two genes were identified as significant DEGs with a 1.2- to 2.2-fold increase in ‘Kefeng 1’, but they were not differentially expressed in ‘NN1138-2’ (Supplementary Table S12, Sheet 1). AtWRKY33 can also interact with an MKS1 protein [AtVQ21, a calcium-binding protein carrying a conserved VQ (Valine-Glutamine) motif], and induce the expression of PAD3 (phytoalexin deficient 3) to enhance the defense response to *P. syringae* pv. *tomato* (*Pst.*) DC3000 (Andreasson et al., 2005; Cheng et al., 2012). The interaction between GmWRKY33 and another MKS1 (GmVQ24, *Glyma.06G124400*) was predicted to be involved in soybean cyst nematode resistance (Huang et al., 2022). Our prediction suggests that the interaction between WRKY33 and MKS1 may play a role in the broad-spectrum resistance of soybean to multiple pathogens.

Pathogen infection causes a series of early signaling events in plants, such as ROS production, activation of MAPKs, induction of plant hormone biosynthesis, and calcium flux (Yang et al., 2022). An interesting finding was that two members of putative WRKY33 (*Glyma.18G208800* and *Glyma.09G280200*) can interact with CML36, and CDPK SK5-like can interact with both CML36 and MAPK7-like. DEGs encoding these five proteins were all up-regulated in ‘Kefeng 1’ but show no differential expression in ‘NN1138-2’. Two other MAPKs (MAPKK9 and MAPKKK3) encoded by *Glyma.07G105700* and *Glyma.04G213000*, which could bind to MAPK7-like, were found with a 1.6-fold and 1.0-fold increased expression levels in ‘Kefeng 1’ (Supplementary Table S12, Sheet 1). All these results demonstrated that MAPKKK/MAPKK/MAPK-WRKY-CML/CDPK might indicate a new complex network in soybean for defense against SMV infection. By summarizing the PPI analysis in this study and combining it with previous reviews on the role of circadian rhythm in plant immunity (Hua, 2013; Wang et al., 2021), we drew a model diagram of soybean resistance response to SMV (Figure 8B).

### 3.10 Validation of candidate DEGs in two cultivars

**Figure 9:**
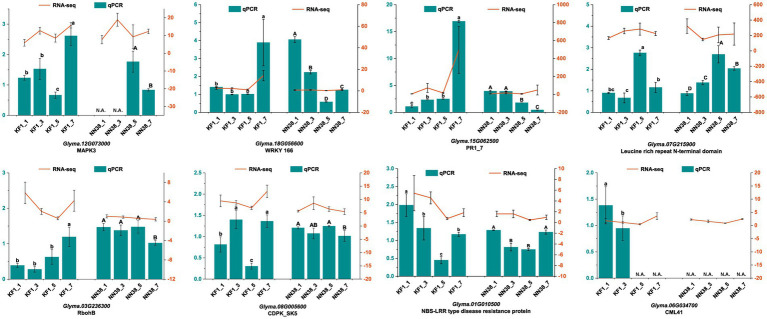
Relative expression levels of eight candidate genes at different time points (1, 3, 5, and 7 dpi) of SMV inoculation in ‘Kefeng 1’ (KF1) and ‘NN1138-2’ (NN38) by qRT-PCR. Left *y*-axis (in cyan) represents the relative expression level of qRT-PCR. Right *y*-axis (in orange) represents the FPKM value of RNA-Seq. N.A. represents the qRT-PCR data that were not available. Error bars represent the standard deviation of the mean relative expression value. Significance between groups was determined by Duncan’s multiple range test (*p* < 0.05).

The accuracy of our transcriptome data was validated by qRT-PCR. Eight DEGs closely associated with resistance to SMV infection were selected as targets based on high FPKM and fold change. The results showed that the expression patterns of DEGs in ‘Kefeng 1’ at 1, 3, 5, and 7 dpi after SMV inoculation were consistent with those calculated by RNA-Seq analysis (Figure 9). These findings support the high reproducibility between replicates of the transcriptome analysis.

## 4 Discussion

Soybean can be infected by more than 20 genera of viruses either naturally or by laboratory inoculation (Loebenstein and Thottappilly, 2003; Hill and Whitham, 2014). Among these viruses, SMV is generally considered to be the most economically important and widespread. In this study, two soybean cultivars with opposite resistance traits and the SMV 6067–1 isolate were used to perform transcriptome sequencing. The results of GO enrichment analysis showed that genes associated with cell wall biogenesis and organization were differentially expressed in both cultivars after SMV inoculation. Among them, 45 members showed up-regulated expression in at least one group of ‘Kefeng 1’ (Supplementary Table S7, Sheet 1), DEGs encoding fasciclin 1 (FAS1) domain-containing proteins (27/45) were the most abundant in this category, and fasciclin-like arabinogalactan proteins (FLAs) are reported to be involved in cell wall biosynthesis and responses to biotic and abiotic stresses (MacMillan et al., 2010, 2015; Zang et al., 2015). Homologs of this class of genes in ‘Kefeng 1’ showed up-regulated expression after SMV infection, but the specific mechanism of resistance and the influence on cell wall metabolism in the process are still unclear. The plant cell wall consists of several enzymes capable of modifying polysaccharides, of which xyloglucan endotransglucosylase/hydrolase (Xet, XTH) is important, as it is essential for wall architecture and elongation (Fry, 1995). XTH/Xet has also been implicated in cell wall loosening and expansion during pathogen infection (Rose et al., 2002). SMV inoculation caused downregulation of the expression of several *XTHs* in both soybean cultivars (Supplementary Table S7, Sheet 2 and Sheet 3), which may be related to inhibition of viral penetration of the cell wall barrier. Similarly, multiple genes encoding pectate lyase (PL) and pectinesterase (PE) were down-regulated after SMV infection in Kefeng 1 and NN1138-2. It is suggested that the synthesis and degree of modification of pectins in the cell wall also affect soybean resistance to SMV.

The KEGG enrichment results highlighted massive genes and multiple metabolic pathways relevant to resistance to SMV. Our study suggests that various proteins involved in the MAPK cascade, hormone signaling, Ca^2+^ signaling, starch and sucrose metabolism, and circadian rhythm, together with transcription factors and R proteins, may form a complex network to regulate the immune response to SMV in soybean, which will be discussed in detail later.

### 4.1 Regulation of TFs in soybean defense response

TFs play a key role in regulating plant responses to biotic stresses and in regulating transcriptional reprogramming associated with stress response (Buscaill and Rivas, 2014; Amorim et al., 2017; Wei and Chen, 2018), which is crucial to elucidate the mechanism in soybean resistance to SMV. Here, we found that four major TF families (bHLH, ERF, MYB, and WRKY) possessed a higher number of DEGs in both cultivars (Figure 3A). Previous studies have shown that members of the WRKY family are particularly important in plant cells, as they are extensively involved in the response to diverse biotic/abiotic stresses and in physiological/developmental processes (Jiang et al., 2015; Sun S. et al., 2023). In this study, ERF and bHLH families have the largest number of differentially expressed TF genes in SMV-inoculated ‘Kefeng 1’ and ‘NN1138-2’, respectively. An interesting finding is that none of the members in MYB family, which plays a broad-spectrum role in plant immune and defense responses (Yu et al., 2023), is enriched in MAPK signaling pathway, plant hormone signal transduction, or plant-pathogen interaction pathway in this study. The possible reason is that this phenomenon depends on the type of plant or virus or the sampling time, i.e., it is not universal. Moreover, the number of differentially expressed *bZIP* genes enriched in these three pathways was the highest (Figures 3B,C). Since this family of TFs is an important component in SA signaling, we believe that SA plays a critical role in soybean defense against SMV.

### 4.2 Plant hormone signaling involved in soybean defense response

Plant hormones are very important signaling molecules associated with the regulation of host-virus interactions (Alazem and Lin, 2015), and previous studies indicated that pathogen invasion will cause changes in endogenous hormone levels in plants (Adie et al., 2007; Robert-Seilaniantz et al., 2007). Until recently, most studies on the role of hormones in plant-pathogen interactions have focused on SA, JA, and ET, which have been recognized as primary defense hormones. However, this study shows that the majority of defense response-related DEGs are associated with IAA signaling (Figures 6A,B).

Previous studies have shown that different DNA-binding AUXIN RESPONSIVE FACTORS (ARFs, also known as auxin-responsive proteins) positively or negatively regulate resistance to *rice dwarf virus* (RDV) infection in *Oryza sativa* (Qin et al., 2020). In two other studies of viral infection in rice, the SP8 protein of *Southern rice black-streaked dwarf virus* (SRBSDV) can interact with OsARF17 and inhibit its ability to bind DNA (Zhang H. et al., 2019). Similarly, the M protein of *rice strip mosaic virus* (RSMV) and the SP2 protein of *rice strip virus* (RSV) also interact with and inhibit OsARF17 (Zhang et al., 2020). Viruses can also manipulate specific ARFs in dicotyledonous plants to influence symptom development. The *tobacco mosaic virus* (TMV) replicase interacts directly with the Arabidopsis PAP1 (PHYTOCHROME-ASSOCIATED PROTEIN 1)/IAA26 (INDOLE-3-ACETIC ACID INDUCIBLE 26), IAA18 and IAA27 proteins through the helicase domain, and enhances TMV pathogenicity (Padmanabhan et al., 2005, 2008). These results suggest that five different viruses have evolved strategies to promote virulence by interacting with specific host proteins and interfering with their different functions in auxin signaling. In the present study, the number of DEGs associated with auxin signaling was the highest among all eight hormones. Among the auxin signaling-related DEGs, those encoding ARFs were the most abundant (37/58; Supplementary Table S9), implying that they play an important role in soybean defense against SMV. In ‘NN1138-2’ seedlings inoculated with SMV, the number of down-regulated *ARF* genes was higher than that in ‘Kefeng 1’. Mock inoculation and SMV infection could decrease the IAA content in the leaves of both soybean cultivars (Figures 6E,F), indicating that mechanical injury and virus infection could interfere with IAA biosynthesis and thus reduce the cell growth rate. How auxin modulates soybean response to SMV requires further experiments to verify. A possible idea in the future is to use the appropriate ARF obtained from RNA-seq as a target protein, screen the SMV protein that can directly interact with it, and further investigate whether the viral protein can interfere with the activity of GmARF, thereby exploring the function of GmARF in viral infection.

Considering only the SMV inoculation condition, the endogenous SA content was consistently increased in both soybean cultivars (Figures 6G,H). The results indicated that SA plays an important role and is a fundamental resistance-related hormone in soybean. We speculate that several bZIP transcription factors connected with SA signaling in Supplementary Table S9 may be involved in the regulation of isochorismate synthase (ICS) activity, thereby promoting SA biosynthesis. The zeatin content of ‘Kefeng 1’ and ‘NN1138-2’ cultivars showed an opposite trend under mock and SMV inoculation conditions (Figures 6I,J), suggesting that it may be involved in the process of soybean resistance and susceptibility to SMV, respectively. Finally, in SMV-infected seedlings, the levels of ABA (Figures 6C,D) and SA peaked at 5 dpi or 7 dpi, indicating that their involvement in the regulation of disease resistance signal transduction takes some time.

### 4.3 Glycometabolism is involved in the SMV pathogenicity

Carbohydrates such as glucose, fructose, sucrose and starch are recognized as sources of carbon and energy (Koch, 2004). They can also interact with other signaling molecules, including hormones, to control plant growth and development (Rolland et al., 2006; Smeekens et al., 2010). Among the DEGs enriched in starch and sucrose metabolism, *BGLU* genes encoding β-glucosidase are involved in several physiological processes as follows: cell wall catabolism (Patro et al., 2014), cell wall lignification (Dos Santos et al., 2019), defense compound activation (Lacchini et al., 2023), plant hormone activation (Han et al., 2020), and release of aromatic volatiles (Sun Y. et al., 2023). As a PR protein belonging to the PR-2 family, one member of GmBGLU (Glyma.15G142400) interacts with a *Phakopsora pachyrhizi* effector, suppressing PTI and promoting virulence (Bueno et al., 2022). In our previous study, the P3 movement protein in SMV was demonstrated to interact with an endo-1,3-β-glucanase to promote viral pathogenicity (Shi et al., 2020). We hypothesize that screening for effector proteins in various pathogens that can interact with BGLU and exploring their functions in the pathogen life cycle will help elucidate the mechanism of pathogenicity induced by BGLU.

In addition, the remaining sugar signaling molecules such as HXK1 may also potentially regulate plant defense. The HXKs are the best studied sugar sensors and have been implicated in the glucose-mediated repression of photosynthetic genes (chlorophyll *a*/*b* binding protein and plastocyanin) (Moore et al., 2003; Cho et al., 2006). Furthermore, HSKs potentially promote the degradation of EIN3, a key transcriptional regulator in ethylene signaling and plant defense (Karve et al., 2012). In this study, two *HXK1* genes (*Glyma.05G226600* and *Glyma.08G033300*) were not differentially expressed in ‘Kefeng 1’ at all sampling points, but were up-regulated at 7 dpi vs. 1 dpi in ‘NN1138-2’ (Supplementary Table S10, Sheet 1). Therefore, how HXK-dependent or HXK-independent metabolic pathways activated by sugar signaling can regulate transcription, translation, and enzyme activity, and then enhance the pathogenicity of SMV in soybean, is an interesting direction of research.

### 4.4 Function of protein–protein interaction modules in soybean defense response against SMV

Plants deploy cell surface and intercellular receptors to sense pathogen infection and initiate immune signaling (Zhou and Zhang, 2020), which are referred to as pattern recognized receptors (PRRs). The MAPK cascade is one of the early signaling events in PTI and ETI (Meng and Zhang, 2013), that can transfer extracellular signals to the intracellular environment, which is also an important regulator of hormonal responses as well as innate immunity (Bi and Zhou, 2017; Thulasi Devendrakumar et al., 2018). This cascade system consists of MAPKKK (MEKK)-MAPKK (MKK)-MAPK (MPK) modules that link upstream receptors and downstream target sensors to form a complete signaling complex capable of recognizing invading pathogens and activating specific defense responses (Zhang and Zhang, 2022). The results of this study indicated that some resistance-response DEGs and susceptibility-response DEGs can be enriched in the MAPK signal pathway simultaneously (Figures 5A,B). Similar report was found in *A. thaliana*, AtMPK4 regulated plant immunity both positively and negatively. The possible reason is that AtMPK4 has three distinct substrates: MKS1, calmodulin-binding receptor-like cytoplasmic kinase 3 (CRCK3) and ARABIDOPSIS SH4-RELATED 3 (ASR3), each of which can interact with different downstream target proteins, to positively or negatively regulate PTI responses (Andreasson et al., 2005; Li et al., 2015; Thulasi Devendrakumar et al., 2018). Therefore, screening the substrates of MAPK in soybean for functional validation will facilitate the investigation of the MAPK signaling cascade in the defense response.

DEGs related to Ca^2+^ signals constitute the largest group in the plant-pathogen interaction pathway (Supplementary Table S10, Sheet 2). CaM and CML proteins are primary Ca^2+^ sensors that control various cellular functions by regulating the activity of different target proteins (Cheval et al., 2013). The effects of various CaMs/CMLs were quite different in pathogen-infected plants. For instance, silencing the expression of *NtCaM13* in tobacco increased susceptibility to viral, bacterial and fungal pathogens, whereas knockdown of *NtCaM1* did not (Takabatake et al., 2007). Another report in *Arabidopsis* suggests that CML37 acts as a positive and CML42 as a negative regulator in defense responses after inoculated with a fungus: *Alternaria brassicicola* (Heyer et al., 2022). In this study, most of the CaM/CML genes enriched in plant-pathogen interaction showed down-regulated expression, while two genes (*Glyma.18G260700* and *Glyma.09G236800*) were up-regulated after viral infection (Figure 7), suggesting that CMLs in soybean act antagonistically in the regulation of induced defense responses. In plants, CaM can bind to some pathogen-induced TFs and induce plant immunity, some of these TFs can link Ca^2+^ signaling and SA, and activate both PTI and ETI (Bari and Jones, 2009). Numerous studies have also suggested that Ca^2+^ is involved in auxin signaling or responses [reviewed in (Vanneste and Friml, 2013)]. It is hypothesized that *SAUR* (*SMALL AUXIN UP RNA*) genes might play a role in linking Ca^2+^ to auxin signaling (Ren and Gray, 2015). Multiple CaMs/CMLs in this study showed opposite expression patterns, implying that they may bind to specific TFs, thereby activating different phytohormone signaling pathways and thus positively or negatively regulating the SMV infestation process. Interestingly, WRKY33 showed different expression patterns between the two soybean cultivars (Supplementary Table S12, Sheet 1). Calcium sensors might promote the binding of WRKY33 to the resistance- or susceptibility-related genes to regulate their transcriptional reprogramming together with the soybean response to SMV. The mechanism of the MEKK3/MKK9/MPK7-WRKY33-CML/CDPK module-mediated response to SMV needs more detailed experimental data to be fully understood.

Many living organisms on Earth, such as bacteria, cyanobacteria, fungi, animals, and plants, have evolved the ability to measure time, using the endogenous oscillator known as the circadian clock, which is critical for the physiological, developmental, and biochemical processes in multiple organisms (Greenham and McClung, 2015; Inoue et al., 2018). It has been reported that the plant’s defensive response to pathogens and pests is also regulated by the circadian clock (Lu et al., 2017). CCA1 (circadian clock associated 1) is a central circadian regulator, *CCA1*-RNAi transgenic *A. thaliana* compromised resistance to the downy mildew while *CCA1* overexpression lines enhanced resistance to this pathogen (Wang W. et al., 2011). One of the *CCA1* genes, namely FKF1, was increased by more than 2-fold in both cultivars after SMV infection (Supplementary Table S12, Sheet 1), suggesting its involvement in the immune response to SMV. Our prediction also indicates that soybean FKF1 has 12 target protein partners for PPI, including CRY1, CRY2, and three PIF transcription factors (PIF4, PIF4A, and PIF4B) (Supplementary Table S12, Sheet 2), suggesting that they may contribute to soybean resistance by forming a modular regulatory mechanism.

Circadian rhythm processes in plants are accompanied by changes in light intensity and quality. The light environment has a major influence on the photosynthesis of plants and their response to pathogens or insect herbivores. Plants have evolved multiple photoreceptor systems, including red (R)/far-red (FR) light-absorbing phytochromes (phyA-phyE in *A. thaliana*), blue/UV-A light-absorbing cryptochromes (CRYs), phototropins (PHOTs), and UV-B-absorbing UVR8 (Ni et al., 1998; Martinez et al., 2018), which regulate various photoreactions through their interactions with downstream target proteins, of which PIF3 is the best characterized (Ni et al., 1998). As one of the pivotal transcription factors involved in photoreceptor-mediated light response, PIFs play important roles in plant defense against necrotrophic pathogens. It has been proposed that PIFs play a role downstream of Phytochrome B (phyB) and participate in a variety of physiological processes, including seed germination, flowering, senescence, and shade avoidance (Kumar et al., 2012; Xie et al., 2017; Zhang R. et al., 2019; Qi et al., 2020). Recently, some results have reported that PIFs redundantly control Arabidopsis defense against *Botrytis cinerea* by modulating the expression of a number of defense-related genes, some of which are involved in ET signaling (Xiang et al., 2020). In the present study, *PIF3* was up-regulated only in ‘Kefeng 1’, can interact with CRY1 and CRY2, and CRY1 can also interact with a candidate R protein (FBXL3), suggesting that PIF3 is involved in soybean resistance to SMV. How PIFs modulate the response of soybean to SMV requires further experiments, and in particular, their downstream target proteins need to be identified.

## 5 Conclusion

Breeding resistant germplasms is the most effective strategy for controlling viral diseases in soybean industry. KEGG enrichment together with PPI analysis revealed that most of the DEGs enriched in plant hormone signal transduction and circadian rhythm pathways, together with transcription factors of ERF, WRKY, and PIF families were the prominent components for resistance responses to SMV. MAPK cascades and Ca^2+^ signaling can positively or negatively regulate soybean resistance to SMV. Through the transcriptome profiling, we demonstrated that the MEKK3/MKK9/MPK7-WRKY33-CML/CDPK module and the PIF-CRY-R protein module can regulate the expression of resistance-related genes. These results will help us understand the regulation of resistance and susceptibility patterns between soybean and SMV, and further functional studies of candidate genes will aid to uncover new control strategies against SMV.

## Data availability statement

The raw datasets presented in this study have been deposited in the NCBI SRA (Sequence Read Archive) database under accession No. PRJNA1024691.

## Author contributions

XYC conceived the project. XYC and XC supervised the work. HL performed most of the experiments with assistance from JL, XY, XC, and XYC. HL, XC, and XYC wrote the manuscript with contributions from all the authors. All authors contributed to the article and approved the submitted version.

## Footnotes

^1^ https://www.soybase.org/GlycineBlastPages/

^2^ https://david.ncifcrf.gov/home.jsp

^3^ http://planttfdb.gao-lab.org/

^4^ https://cn.string-db.org/

^5^ http://www.ncbi.nlm.nih.gov/tools/primer-blast
