# Supplementary material for: Comparative transcriptome analysis reveals key pathways and regulatory networks in early resistance of Glycine max to soybean mosaic virus
Source: Front Microbiol. 2023 Oct 19;14:1241076. doi: 10.3389/fmicb.2023.1241076 (PMC10687721; doi:10.3389/fmicb.2023.1241076)
Supplement: Supplementary file 1 [file Data_Sheet_1.zip › Supplementary Figures.pdf]

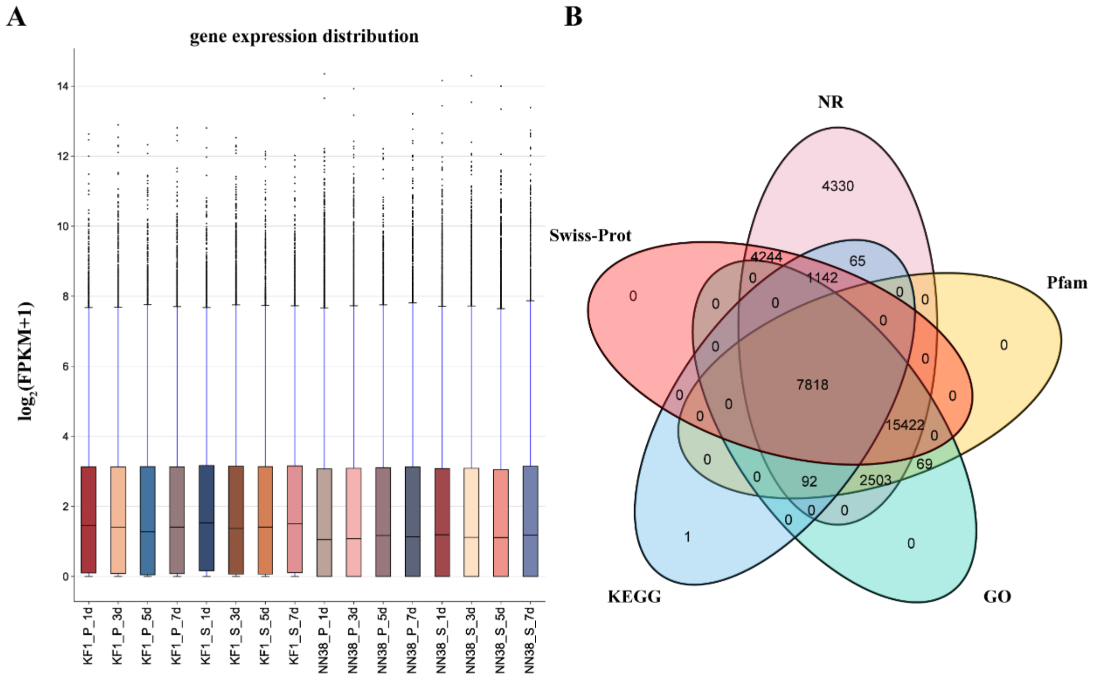

Supplementary Figure S1. Transcriptome sequencing and quality assessment. (**A**) Boxes show the gene expression profile of each treated sample. KF1 is short for Kefeng 1, NN38 is short for NN1138-2, P is short for PBS (mock) inoculation, and S is short for SMV inoculation. (**B**) Venn diagram showing the annotated information of all genes from the NR, Pfam, GO, KO, and Swiss-Prot databases.

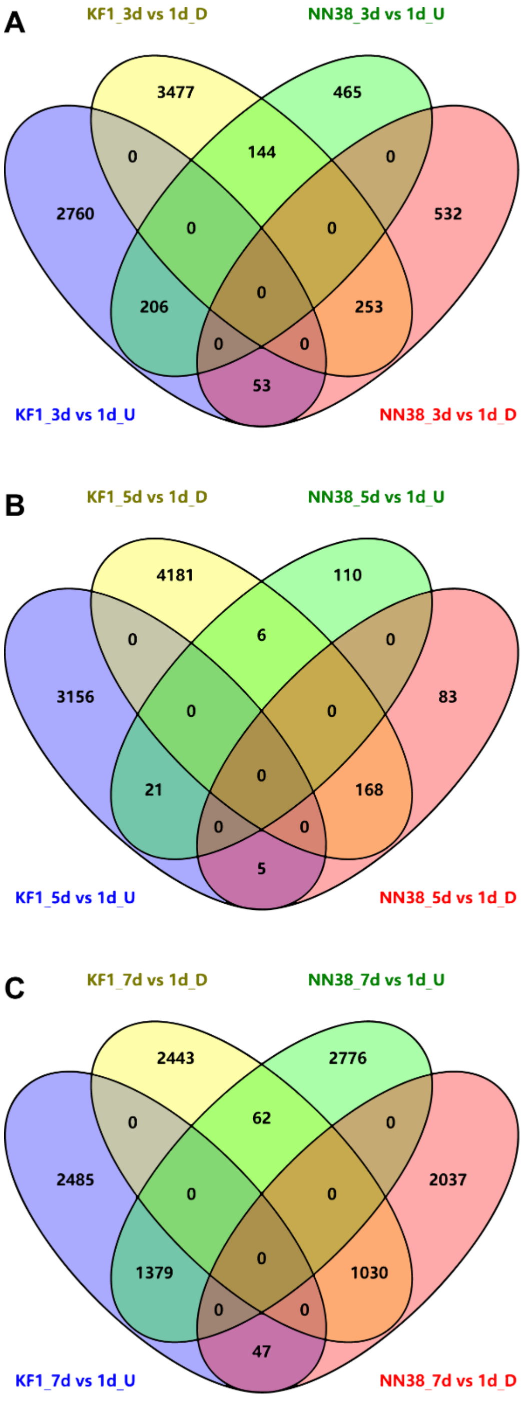

Supplementary Figure S2. Venn analysis of contrasting expression patterns of DEGs between Kefeng 1 (KF1) and NN1138-2 (NN38) at the same time point. (**A**) 3 dpi vs 1 dpi; (**B**) 5 dpi vs 1 dpi; (**C**) 7 dpi vs 1 dpi. U: up-regulated DEGs; D: down-regulated DEGs.

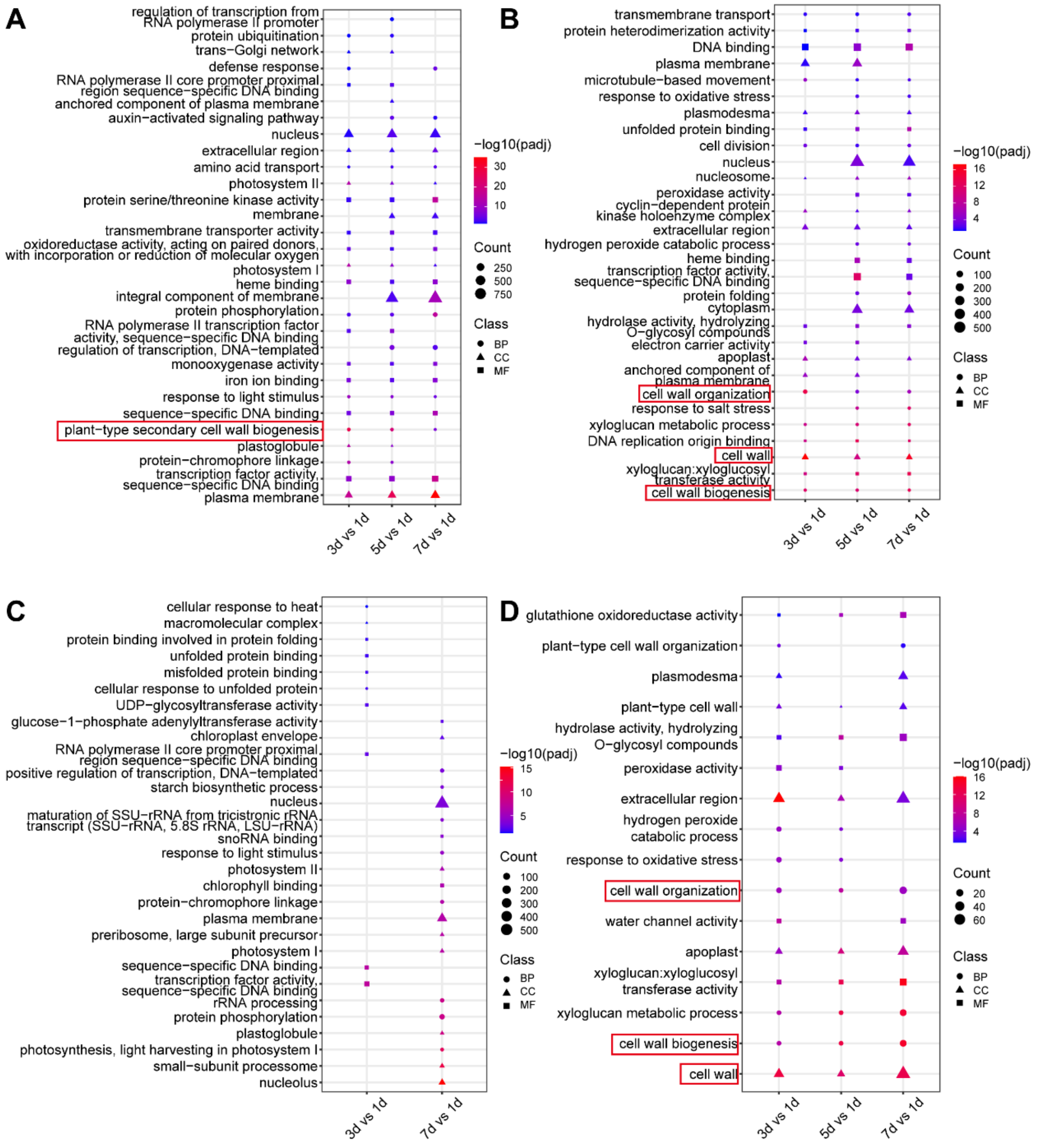

Supplementary Figure S3. GO enrichment analysis of DEGs in different comparisons. (**A**) Classification of the top 30 enriched GO terms associated with up-regulated DEGs in Kefeng 1. (**B**) Classification of the top 30 enriched GO terms associated with down-regulated DEGs in Kefeng 1. (**C**) Classification of the top 30 enriched GO terms associated with up-regulated DEGs in NN1138-2. (**D**) Classification of all the enriched GO terms associated with down-regulated DEGs in NN1138-2. GO terms in red boxes relate to metabolisms in plant cell walls.
